# Audio self-supervised learning: A survey

**DOI:** 10.1016/j.patter.2022.100616

**Published:** 2022-12-09

**Authors:** Shuo Liu, Adria Mallol-Ragolta, Emilia Parada-Cabaleiro, Kun Qian, Xin Jing, Alexander Kathan, Bin Hu, Björn W. Schuller

**Affiliations:** 1 Chair of Embedded Intelligence for Health Care & Wellbeing, University of Augsburg, 86159 Augsburg, Germany; 2 Institute of Computational Perception, Johannes Kepler University Linz, 4040 Linz, Austria; 3 School of Medical Technology, Beijing Institute of Technology, Beijing 100081, China; 4 GLAM – the Group on Language, Audio, & Music, Imperial College London, London SW7 2AZ, UK

**Keywords:** self-supervised learning, audio and speech processing, multi-modal SSL, representation learning, unsupervised learning

## Abstract

Similar to humans’ cognitive ability to generalize knowledge and skills, self-supervised learning (SSL) targets discovering general representations from large-scale data. This, through the use of pre-trained SSL models for downstream tasks, alleviates the need for human annotation, which is an expensive and time-consuming task. Its success in the fields of computer vision and natural language processing have prompted its recent adoption into the field of audio and speech processing. Comprehensive reviews summarizing the knowledge in audio SSL are currently missing. To fill this gap, we provide an overview of the SSL methods used for audio and speech processing applications. Herein, we also summarize the empirical works that exploit audio modality in multi-modal SSL frameworks and the existing suitable benchmarks to evaluate the power of SSL in the computer audition domain. Finally, we discuss some open problems and point out the future directions in the development of audio SSL.

## The bigger picture

Several current review studies seek to provide the scientific community with an overview of the existing literature on self-supervised learning (SSL). However, these studies clearly favor computer vision (CV) and natural language processing (NLP) owing to their widespread use in these domains. The success of SSL in these fields has inspired its incorporation into audio processing. Therefore, the purpose of this survey is to present an overview of the SSL techniques used in audio and speech processing applications. In addition, we summarize the empirical research that uses the audio modality in multi-modal SSL frameworks, as well as the available benchmarks that can be used to assess the effectiveness of SSL in the area of computer audition.

## Teaser

Recent research has shown an ever-growing interest in applying SSL to audio and speech processing. As this rapid emerging field has not yet been thoroughly explored, we provide a survey on SSL with a focus on the recent advancements, including for the first time an overview of SSL in audio within unified frameworks. This review is intended to benefit practitioners, both beginners and more experienced researchers, who are interested in the use of SSL for audio signal processing.

## Introduction

According to Piaget’s theory of cognitive development,^1^^,^^2^ from their birth up to approximately 18 months, children acquire knowledge from sensory and motor experiences. During this stage, i.e., the “sensorimotor” stage, through basic actions such as sucking, grasping, looking, and listening, the early representational thought emerges.^3^ Along with the acquisition of knowledge, over the different developmental stages until the last one, i.e., the “formal operational” (adolescence and adulthood) stage, children’s reasoning progressively moves toward the acquisition of abstract ideas and the use of deductive logic, i.e., subtracting specific information from a general principle.^4^ During this process, in order to understand the world, the so-called “schemas,” i.e., higher-order cognitive structures that have been hypothesized to underlie many aspects of human knowledge and skill,^5^ emerge. According to Piaget, child development is interpreted trough an equilibration mechanism that explains how new information is balanced according to old knowledge. Equilibration involves “assimilation” (the process of taking in new information to fit in with the pre-existing schemas) and “accommodation” (the process of modifying the pre-existing schemas as a result of new information).^1^^,^^6^ In this view, learning is possible if complex structures are based on simpler ones, i.e., when a natural development between structures exists instead of a simple external reinforcement.^1^ Indeed, the interesting aspect of learning (and one of the main goals in education) is to create dynamic structures that can lead to generalization, i.e., the ability to apply learned knowledge and skills for understanding in a different context. This is known as “transfer of learning.”^7^ Similar to the cognitive process of developing dynamic structures with the capability of generalization, the self-supervised learning (SSL) paradigm has been presented^8^^,^^9^^,^^10^^,^^11^—a machine- and deep-learning technique that has rapidly evolved in the last years. SSL targets at learning a model that is able to produce universal representations. This is approached by first solving some pretext tasks (also known as upstream tasks in literature), i.e., a procedure that, similarly to the sensorimotor stage, enables someone to artificially learn representations directly from the data attributes without the need for human annotations.^12^ Then, with a pre-trained model generated on the pretext task, feature representations are extracted to understand new data, i.e., similarly to cognitive development, a pre-trained model (previous knowledge) can be used through generalization to understand a new context, a process known as downstream task.^13^

SSL mitigates two difficulties that currently limit the application of deep learning: the need of human annotations in representation learning and the difficulty in designing effective network architectures for specific tasks. First, the current success of deep learning reckons on big data, which typically consume uninhibited human efforts in annotations. This faces the issue of annotation bias as well as the fact that annotation procedures often cannot optimally preserve data privacy. As SSL learns representations from the data itself without the need of labels^14^ (sometimes creates pseudo-labels for self-supervision), it overtakes the challenges derived by the use of human annotations. Moreover, many works, such as Chen et al.,^15^ have shown that much less labeled data are needed to fine-tune an SSL model for downstream tasks in order to achieve similar (or even better) performance compared with the conventional supervised-learning setting. Second, as long as the pretext model can generate proper representations of the data, these can be used for multiple downstream tasks, reducing, at the same time, the difficulty in designing reliable downstream models. For instance, a multi-layer perceptron (MLP) is commonly used for this step, reaching state-of-the-art results for different research areas in artificial intelligence. As the main effort of SSL concentrates on the development of well-trained upstream models, it guarantees the extraction of data representations with a sufficient level of generalization and distinctiveness. Furthermore, as a way to increase distinctiveness of the learned representations, when solving pretext tasks, negative examples can be additionally provided in order to contrast the target sample with negative examples.^16^ This process formalizes the SSL into a contrastive learning framework.^17^^,^^18^^,^^19^^,^^20^

The fitness of upstream and downstream tasks, i.e., how much the knowledge learned from pretext tasks is applicable to downstream tasks, is partially determined by the data relevance used in both steps. From a cognitive point of view, this is comparable to the aforementioned transfer of learning, as a speaker of a given language would find it easier to learn a related language (near transfer) than an unrelated one (far transfer). Thus, near transfer of knowledge is expected to ensure that the downstream tasks particularly benefit from the upstream training. However, far transfer may also occur. Therefore, downstream tasks that use data from a different domain can still benefit from learning representations of sufficient generalizability. The versatility of SSL has yielded to a superior performance in several research fields, such as natural language processing (NLP)^21^ and computer vision (CV),^8^^,^^11^ as well as in a variety of deep-learning methods, e.g., graphical neural networks^22^ and reinforcement learning,^23^ to name a few. Nevertheless, processing audio sources increases further the difficulty of applying deep-learning methods as in the real world, this modality is typically characterized by many uncertainties. Speech, for instance, due to within- and cross-speaker variations, such as those produced by disfluencies, as well as differences in language, acoustic environments, or recording setups, usually presents considerable variability. This makes it difficult to deduce relevant latent structures without taking into account any supervision guidance. In addition, unlike for images, overlapping noise is typical of recordings. Through its masking properties, surrounding noise limits (and even impede) understanding, in some cases distorting the spectrogram of the audio content of interest. Indeed, as each pixel (time-frequency bin) of the spectrogram can be deteriorated, noise reduction and removal is still an open challenge in the field.^24^ Similarly, compared with NLP tasks, which (despite their inherent difficulties) process texts that are comprised of a limited amount of possible words and characters, the infinite possibilities of audio that represent the same meaning create more uncertainties in audio understanding. These facts indicate the problems that need to be especially considered when applying SSL to audio and speech processing.

Specifically, a proper SSL model should be able to extract representations that are (1) distributed, i.e., more expressive as the dimensionality increases; (2) abstract, i.e., aggregate more abstract features that are invariant to local changes in the input; and (3) disentangled, i.e., each factor of the representation vector should be interpretable.^9^ As SSL requires from a model both generalization and discrimination (in parallel), using SSL for audio processing becomes particularly challenging. Although several survey articles aimed to give an overview of the existing literature on SSL that have been presented to the research community, due to the prominent use of SSL in CV and NLP, these works show a clear bias toward these two fields.^8^^,^^11^^,^^21^ However, despite the challenges, recent research has shown an always increasing interest in applying SSL to audio sources. As this rapidly developing area has not been systematically investigated yet, to fill this gap, we present a survey on SSL with a special emphasis on the recent progress by including, for the first time, an overview of SSL in audio within unified frameworks. By providing an overview of the existing techniques as well as a disambiguation between approaches, this work is especially thought to support practitioners, both beginners and more experienced researchers, interested in the use of SSL for audio signal processing.

The rest of the manuscript is organized as follows. We first give a general overview of SSL, which mainly explores approaches for CV and NLP domains. This section covers different components of the learning framework, including input data format, data augmentation, network structures, the construction of training objectives, and the description of the basic blocks and operations that lead to its success. Then, we assess how these frameworks can be related to audio processing by considering the commonalities and differences between audio and other data formats such as video or text. Next, SSL approaches exploiting audio as one of the modalities will be discussed. Additionally, we summarize the downstream tasks considered in the literature and list the databases and benchmarks that are used for evaluating the performance of pretext tasks. At last, we discuss several aspects of SSL, including its relations to and differences from other similar deep-learning techniques, before drawing a conclusion and pointing out potential research directions.

## SSL: A general overview

SSL aims at learning latent representations from large-scale data by solving designed pretext tasks rather than using human annotations. To this end, different views of an object, which are of high natural correlation, are created. Based on the views, an SSL model is trained to generalize, to some extent, the representations of the object in a latent high-dimensional space.^25^^,^^26^ By contrasting the representations of the same object to other objects (defined as negative samples) in training, a contrastive SSL model is expected to produce representations that are of better distinctiveness.

In the following, we will first introduce SSL frameworks, distinguishing their respective principles of model construction, allocation, and training objectives by having a particular emphasis on the approaches used to create pseudo-labels as supervisory signals for training the model. Having the frameworks in mind, we will also describe more advanced approaches able to produce different views of an object, as well as their methods to generate proper negatives for training contrastive SSL models. We will end this section by summarizing the approaches used to fit the SSL model for processing sequential data, such as video and text. Considering the characteristics of different kinds of sequential data and learning objectives, additional ways to generate different views, and negatives if needed, for training SSL models will also be presented. A summary table of the typical SSL methods is shown in Table 1.

**Table 1:** An overview of the recent typical self-supervised learning methods

Model	FoS	Framework	Encoder	Pseudo-labels	Loss	Negative samples	
						Source	Strategy
TCN embedding^45^ (2018)	CV	(d)	inception network + CNN	different but simultaneous viewpoints	triplet loss	images of different time	end to end
SimCLR^15^ (2020)	CV	(d)	ResNet	data augmentation	NT-Xent loss	other images	end to end
SimCLR v.2^193^ (2020, semi)	CV	(d)	variants of ResNet	data augmentation	NT-Xent loss	other images	end to end
MoCo^29^ (2020)	CV	(d)	ResNet	data augmentation	InfoNCE loss	other images	momentum
MoCo v.2^194^ (2020)	CV	(d)	ResNet	data augmentation	InfoNCE loss	other images	momentum
MoCo v.3^168^(2021)	CV	(d)	vision transformers	data augmentation	InfoNCE loss	other images	end to end
RotNet^32^ (2018)	CV	(a)	ConvNet	rotation directions	prediction loss	–	–
Colorization^31^(2017)	CV	(a)	AlexNet, VGG-16, ResNet-152	color of missing patch	regression loss, KL divergence	–	–
DIM^46^(2018)	CV	(d)	–	–	JSD, DV, or InfoNCE loss	–	end to end
Word2Vec^64^ (2019)	NLP	(a)	auto-encoder	context words	prediction loss	–	–
BERT^67^ (2019)	NLP	(a)	MPC	masked words	prediction loss	–	–
ALBERT^36^(2020)	NLP	(a)	MPC	masked words, sentence order	prediction loss	–	–
BYOL^54^ (2020)	CV	(b)	ResNet	data augmentation	MSE loss	–	–
Barlow Twins^55^ (2021)	CV	(b)	ResNet	data augmentation	Equation 3	–	–
SimSiam^50^ (2021)	CV	(b)	ResNet	data augmentation	negative cosine similarity	–	–
DeepCluster^57^(2018)	CV	(c)	AlexNet, VGG-16	clustering centroids	negative log-softmax loss	–	–
Local Aggregation^59^ (2019)	CV	(c)	AlexNet, VGG-16	soft-clustering centroids	negative log-softmax loss	–	–
SwAV^60^ (2020)	CV	(c)	variants of ResNet-50	online-clustering centroids	modified cross-entropy	–	end to end
CPC^42^ (2018)	CV, audio NLP	(d)	APC	–	InfoNCE loss	other images	end to end
CPC v.2^71^ (2020)	CV	(d)	APC	–	InfoNCE loss	other images	end to end

**Figure 1:**
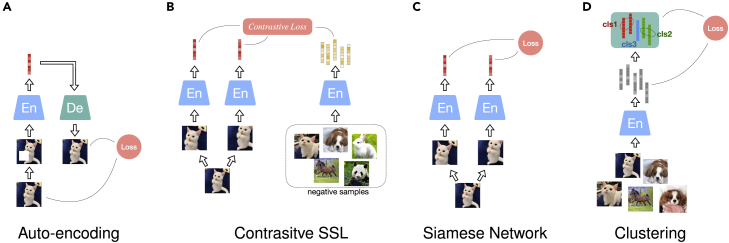
SSL frameworks Predictive SSL frameworks (A–C) and contrastive SSL framework (D). For each framework, the diagram shows the components, including pseudo-labels, that are used to construct training objectives. (A) Auto-encoding contains an encoder (En) and a decoder (De). The En learns representations from a distorted signal input, while the De aims at recovering the clean signal from the learned representations. (B) A Siamese network processes two views of the same data point, hence the latent representation of one sub-network is seen as pseudo-label of the other sub-network. (C) Clustering is applied for grouping the learnt representations—the clustering centroids are used as pseudo-labels for training. (D) Contrastive SSL constructs the contrastive loss through negative samples.

Model, field of study (FoS), type of frameworks (referring to Figure 1), encoder, pseudo-labels, and loss, as well as source and strategy for the negative samples, are given. “Other images,” in the source column, indicates other images of the mini-batch.

### SSL frameworks

We introduce the general SSL frameworks, depicted in Figure 1, without specifying the neural networks. These frameworks exhibit the most typical pretext tasks that need to be solved in training an SSL model without using human annotations on data but with the supervisory signals, i.e., pseudo-labels, that originate from the data itself.

To easily demonstrate the following SSL frameworks, the model inputs can be supposed to be in the ideally simplest form. Specifically, two deformations of an image are regarded as the image’s two views when needed. For contrastive SSL, negative samples are requested and supposed to stem from other distinct images.

#### Auto-encoding

The basic form of predictive SSL models uses auto-encoders,^27^ as depicted in Figure 1A. A standard auto-encoder learns a compressed latent embedding that represents the input of the encoder and expects to reconstruct the original input from the latent representation, i.e., the decoder output. The dimensionality of the latent representation must be carefully designed as it determines the representation reliability. When setting a too-large latent dimensionality, an auto-encoder risks learning an identity function, i.e., maps the input directly to the output and, hence, becomes useless. Various techniques to prevent auto-encoders from learning an identity function do exist, e.g., denoising auto-encoders,^28^ which partially corrupt the input data by randomly zeroing some input values and are trained to recover the original undistorted input. For the denoising to be successful, the model’s ability to retrieve useful high-level representations becomes essential. The zero-out step can be replaced by other data augmentation techniques, such as geometric transformations including cropping,^15^^,^^29^ rotation,^30^ reordering, colorization,^31^ and distortion,^15^ to name a few, which often appear as methods to create different views of data in SSL studies.

The auto-encoding framework presents the fundamental form of predictive models for SSL, including language models and acoustic models based on auto-regressive predictive coding (APC) and masked predictive coding (MPC), which are introduced in the sections semantic representation for sequential data and audio SSL, respectively. Other auto-encoding predictive models for SSL also aim to predict the relative position of signal parts,^32^^,^^33^ including solving a jigsaw puzzle^34^^,^^35^ or reordering the pieces of a shuffled sequence input.^36^^,^^37^^,^^38^

#### Contrastive SSL

Contrastive SSL is typically performed in the context of a triplet network, as shown in Figure 1B. Given a data point, the model requires inputs of its different views and additional negative samples. The encoded representations of the given data serve as the positive pseudo-labels to each other, while the negative samples provide the opposite pseudo-labels. Theoretical analysis has proven that when two views result in redundant information of the label, applying linear projections to the learned representations can guarantee the performance on downstream prediction tasks.^20^ This proof indicates that SSL can produce high-quality representations from the multi-views of a data point and guarantees prediction performance with simple downstream models. While the representations of the given data with different views are attracted closest during training, the distinctiveness of these representations can be improved by contrasting them with the representations of the negative samples.

The effect of contrastive losses can be decisive for the performance of an SSL model. Some such losses were presented to solve supervised-learning problems. For a given anchor *x*, the positive sample *x*^+^ is selected from the same class as the anchor, and the negatives *x*^−^ are from different classes. To keep the same notation in the formulations herein, *x* and $x^{+}$ should be broadly understood as two views of the same data, and the negatives originated from different data. Hereby, we go through the typical types of contrastive loss before going through the contrastive SSL methods in the literature.

##### Contrastive loss

In early versions of contrastive loss, an anchor is paired with only one positive and one negative sample, leading to a positive pair and a negative pair. Recent works have found that it is more effective to take into consideration multiple positive and negative pairs in the training objectives.

Max margin contrastive loss, designed for deep metric learning,^39^ takes a pair of inputs and minimizes the embedding distance when they are from the same class and maximizes it otherwise. More formally, it learns an encoder *f* that minimizes

(Equation 1) $$\begin{array}{l} L\left(x,x^{+}\right)=\sum_{x\in X}{\left\|f\left(x\right)-f\left(x^{+}\right)\right\|}_{2}^{2}\\ L\left(x,x^{-}\right)=\sum_{x\in X}\text{max}{\left(0,\varepsilon -{\left\|f\left(x\right)-f\left(x^{-}\right)\right\|}_{2}\right)}^{2}\;, \end{array}$$

where X stands for a batch of samples including *x* and ε is a hyper-parameter that defines the lowest offset distance between representations of different samples.

Triplet loss^40^ combines the two separate optimization objectives into a single formulation,

(Equation 2) $$L\left(x,x^{+},x^{-}\right)=\sum_{x\in X}\;\text{max}\left(0,{\left\|f\left(x\right)-f\left(x^{+}\right)\right\|}_{2}^{2}-{\left\|f\left(x\right)-f\left(x^{-}\right)\right\|}_{2}^{2}+\varepsilon \right),$$

indicating the beginning prototype of contrastive learning.

Multi-class N-pair loss^41^ generalizes the triplet loss, enabling the contrasts with multiple negative samples. The definition of this loss is on multiple input pairs. It is formulated similarly to the softmax loss:

(Equation 3) $$\begin{array}{l} L\left(x,x^{+},x_{n\in \left[1\text{,}\;N-1\right]}^{-}\right)=\text{log}\left(1+\sum_{n=1}^{N-1}e^{f{\left(x\right)}^{\text{T}}f\left(x_{n}^{-}\right)-f{\left(x\right)}^{\text{T}}f\left(x^{+}\right)}\right)\\ \;=-\text{log}\frac{e^{f{\left(x\right)}^{\text{T}}f\left(x^{+}\right)}}{e^{f{\left(x\right)}^{\text{T}}f\left(x^{+}\right)}+\sum_{n=1}^{N-1}e^{f{\left(x\right)}^{\text{T}}f\left(x_{n}^{-}\right)}}. \end{array}$$

The InfoNCE^42^ objective, inspired by noise-contrastive estimation (NCE), is also known as NT-Xent loss, short for normalized temperature-scaled cross-entropy loss. It introduces an additional temperature parameter for controlling the penalty on the effect of negative samples, similarly as ε in Equations 1 and 2:

(Equation 4) $$L\left(x,x^{+},x_{n\in \left[1\text{,}\;N-1\right]}^{-}\right)=-\text{log}\frac{e^{f{\left(x\right)}^{\text{T}}f\left(x^{+}\right)/\tau }}{e^{f{\left(x\right)}^{\text{T}}f\left(x^{+}\right)/\tau }+\sum_{n=1}^{N-1}e^{f{\left(x\right)}^{\text{T}}f\left(x_{n}^{-}\right)/\tau }}.$$

Its denominator terms contain one positive and N−1 negative samples. Hence, we can construct a softmax classifier that is optimized using cross-entropy loss for *N* classes. The classifier assigns large and small values to the positive and negative examples, respectively. In this regard, InfoNCE can be seen as using categorical cross-entropy loss to identify a positive sample within a set of (unrelated) noise samples. Contrasting the distance of a data point to its positive samples with respect to the one to its negative samples prevents the model from falling into representational collapse. To analyze this effect, we can split it into two parts:

(Equation 5) $$L=E\left[-\text{log}\frac{e^{f{\left(x\right)}^{\text{T}}f\left(x^{+}\right)/\tau }}{e^{f{\left(x\right)}^{\text{T}}f\left(x^{+}\right)/\tau }+\sum_{n=1}^{N-1}e^{f{\left(x\right)}^{\text{T}}f\left(x_{n}^{-}\right)/\tau }}\right]=\underset{\text{alignment}}{\underset{︸}{E\left[-f{\left(x\right)}^{\text{T}}f\left(x^{+}\right)/\tau \right]}}+\underset{\text{uniformity}}{\underset{︸}{E\left[\text{log}\left(e^{f{\left(x\right)}^{\text{T}}f\left(x^{+}\right)/\tau }+\sum_{n=1}^{N-1}e^{f{\left(x\right)}^{\text{T}}f\left(x_{n}^{-}\right)/\tau }\right)\right]}},$$

where the “alignment” term targets at maximizing the similarity between the learned embeddings of the positive pairs. Then, the “uniformity” term helps the contrastive learning to learn separable features by maximal-uniformly distributing the embeddings on a unit sphere given the normalization condition. Both terms are crucial to the downstream tasks according to Wang and Isola.^43^ Different from an instance discrimination objective, which pushes all different instances apart and considers no underlying relations between samples, the design of contrastive loss requires a proper temperature coefficient τ that finds a balance between learning separable features and at the same time providing some degree of tolerance to the closeness of semantically similar samples. A too-small τ loses the tolerance to group the similar input samples and hence may break the underlying semantic structure, thus harming the learned features for its use in downstream tasks. The effect of the temperature parameter is similar as the margin value set in Equation 1, which has been investigated in detail by Liu et al.^44^

In Wang and Liu,^16^ Wang suggests adjusting the alignment and uniformity loss to

(Equation 6) $$\begin{array}{c} L_{\text{align}}=E\left[{\left\|f\left(x\right)-f\left(x^{+}\right)\right\|}_{2}^{\alpha }\right]\\ L_{\text{uniform}}=\text{log}E\left[e^{-t{\left\|f\left(x\right)-f\left(x^{\text{∗}}\right)\right\|}_{2}^{2}}\right], \end{array}$$

indicating that both terms should be minimized simultaneously. Maintaining a good balance between these two terms has been found to be more effective than standard contrastive loss.

Two representative SSL architectures that are trained by using contrastive loss are SimCLR^15^ and momentum contrast (MoCo).^29^ SimCLR^15^ exploits several different data-augmentation techniques for transforming an input image, including random cropping, resizing, color distortions, and Gaussian blur. The transformed images are then coded into representations using ResNet. After going through projection heads built on Dense-ReLU-Dense structure, NT-Xent is used as objective function for SSL. The authors of SimCLR emphasized the importance of “scaling up,” i.e., using a larger batch size and a deeper and wider network, as well as training for longer epochs, in order to guarantee the success of the method. Unlike SimCLR, which uses only one encoder *f*, MoCo^29^ exploits an additional momentum encoder $f_{m}$. The encoder and momentum encoder, sharing the same architecture and being identically initialized, process two views of an image. The method also applies contrastive loss, where the negative samples are provided by previous batches. For this, representations of previous samples are stored into a queue during training. Representations from a new batch are pushed into the queue after training, and old representations are excluded. The encoder is updated by applying back propagation as in SimCLR, while the momentum encoder is updated by linear interpolation of the two encoders, as introduced in Equation 7. The momentum parameter is set to ξ=0.999 by default, meaning that the update of the momentum encoder is much slower. However, the update mode of the momentum encoder avoids back propagation, which can hence increase the number of negative samples for training. The synchronize update of the encoder and momentum encoder also solves the problem of inconsistently encoded representations happening in works using memory bank.^29^ Posterior architectures such as MoCo v.2^30^ integrate the effective components presented in SimCLR. MoCo v.2 incorporates stronger data-augmentation techniques, i.e., using an additional Gaussian deblur method and a larger batch size. Moreover, its projection head layer is increased as a two-layer MLP for both the encoder and momentum encoder. Similarly, SimCLR v.2^28^ upgrades the system proposed in SimCLR by scaling up the model size from ResNet-50 to ResNet-152 and improving the depth of the projection head. In addition, the authors leave one projection layer for fine-tuning on semi-supervised tasks, aiming to learn from few labeled examples while making the best use of a large amount of unlabeled data. Furthermore, in order to efficiently provide a large number of negative samples for training, the idea of memory mechanism used in MoCo v.2 is employed in SimCLR v.2, too. Differently, the latest MoCo v.3^31^ removes the memory queue with the cost of requiring a bigger batch size. In addition, it applies a prediction layer after the projection head, similarly as proposed in bootstrap your own latent (BYOL; introduced in non-contrastive SSL), which successfully improves the representation capability.

Multiple views of the same context can also be obtained by recording it using multiple sensors, such as multiple cameras shooting a scene from different angles.^45^ More broadly, these views can be of different modalities. The mutual supervision of these modalities provides the basis to perform multi-modal SSL, which will be introduced in multi-modal audio representation. Considering the spatial coherence and consistency in data, local features of different patches of an image can be considered as multiple views of the same type of data. In this case, an SSL approach aims to maximize the mutual information between local features and global representation, which aggregates the information of the entire context global information.^46^ An SSL model then learns to represent local features by capturing meaningful information relevant to the aggregated global representation. Deep InfoMax^46^ codes an image into a global context vector and contrasts its distance to the spatial patches of the same image against the distance to spatial patches of different images. However, Tschannen et al.^47^ provide some empirical evidence indicating that the contrastive loss is not only attributed to mutual information. Similarly, Poole et al.^48^ investigates the effect of the redundancy in two views of the positive pair, suggesting that the views with less mutual information should be selected for training. The idea is to compress the redundancy in the embeddings of the two views that are not relevant to the labels.

#### Non-contrastive SSL

Discarding the use of negative samples in SSL, the framework turns into a Siamese architecture as shown in Figure 1C. A Siamese network consists of two parallel sub-networks; each can process a view of a data sample. Considering the natural similarity between the two views of the same sample, the encoded representations in the high-dimensional latent space should be close to each other. Hence, during training, the representations from one sub-network can be seen as the training target, i.e., pseudo-labels, for the other sub-network. The neural encoders of both sub-networks share the same or similar architecture—their parameters can be shared or independent. However, training such a symmetric model without using negative samples is prone to mode collapse, i.e., when the model’s output is very similar (or even identical) for different inputs. To avoid trivial solutions, asymmetry configurations in the architecture need to be considered for the two branches of the Siamese network. Alternatively, for identical branches, these should be trained asynchronously.

**Figure 2:**
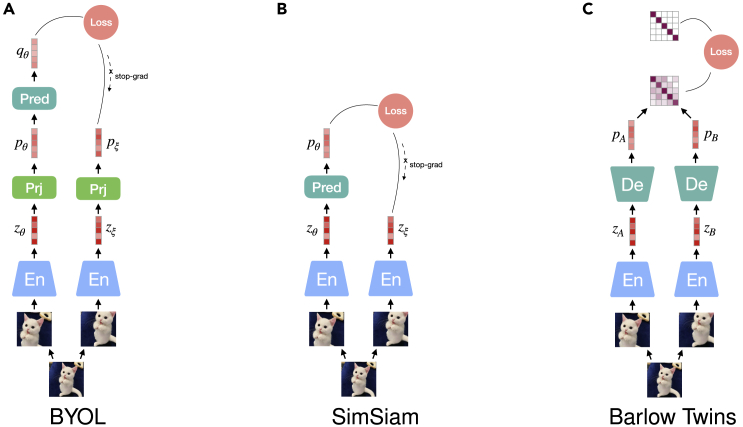
Diagrams for predictive models using Siamese architectures SimSiam^50^ (shown in B) simplifies the structure of BYOL (A) by removing its projection layers used in both sub-networks. Unlike BYOL, the two branches of SimSiam share their parameters, and therefore, it is also seen as an approach of SimCLR without using negative samples. Its success in preventing the model from collapsing into trivial representations can be attributed to two essential factors, i.e., the extra learnable predictor and a stop-gradient operation.^51^

BYOL^49^ trains two sub-networks separately denoted as an online network and a target network, as shown in Figure 2A. Both sub-networks contain an encoder *f* and a projection layer *g*, and the online network has an additional predictor layer *p* build on MLP. The online network is learning to equate its predicted representation and the pseudo-labels, i.e., the projected embedding from the target network. To get rid of mode collapse, the two networks are asynchronously optimized in an iterative way. The target network is randomly initialized, and then its parameters are updated using an exponential moving average (EMA) strategy during training, similar to that presented in MoCo^29^ and defined as

(Equation 7) ξ←τξ+(1−τ)θ,

where *θ* and *ξ* stand for the parameters of the online and target networks and τ∈[0,1] is a given decay rate for updating. The online network follows the guidance of the slowly updated target network and is optimized by minimizing the mean-squared error (MSE) between the two network outputs:

(Equation 8) $$L={\left\|\bar{q_{\theta }}-\bar{p_{\xi }}\right\|}_{2}^{2}=2-2\frac{\left\langle q_{\theta },p_{\xi }\right\rangle }{{\left\|q_{\theta }\right\|}_{2}\cdot {\left\|z_{\xi }\right\|}_{2}},$$

where $\bar{q_{\theta }}$ and $\bar{p_{\xi }}$ are L2-normalized $q_{\theta }$ and $p_{\xi }$, i.e., $q_{\theta }/||q_{\theta }|{|}_{2}$ and $p_{\xi }/||p_{\xi }|{|}_{2}$. The two views are exchanged once as the input of online and target networks to create a symmetric loss, denoted as $\tilde{L}$, leading to a complete training loss of $L+\tilde{L}$. The slow update of the target network progressively aggregates the parameters from the online network. This enables the production of more stable representations, which are used as the guidance to train the online network, thus progressively yielding better representations. As updating the online parameters is a sensitive procedure that requires very careful fine-tuning, in order to avoid mode collapse, the authors additionally exploit LARS^52^ as an optimizer to update the parameters of different layers with different strengths, guiding the model to gradually reach a meaningful convergence.

In addition, the theoretical analysis and experimental study in Tian et al.^51^ has raised two additional suggestions for training non-contrastive SSL models like BYOL and SimSiam. First, the predictor is expected to be updated with a moderately larger learning rate or more frequently (to some degree) than the rest of the network so that mode collapse can be better avoided. Still, a too-frequent update or a too-large learning rate may impair the learning of an optimal predictor capable of achieving a minimal L2 error between the outputs of network twins, hence making it unable to guarantee the quality of the learned representations. Second, applying weight decay has been shown to be very helpful in achieving stable convergence. Although the use of batch normalization^53^ was hypothesized to be crucial for preventing collapse in BYOL (https://generallyintelligent.ai/blog/2020-08-24-understanding-self-supervised-contrastive-learning/), in previous work,^54^ batch normalization has been successfully replaced with group normalization and weight standardization, thus refuting the need of batch statistics for BYOL.

Barlow Twins (BT)^55^ is a neural network that holds a symmetric structure with its two branches, as depicted in Figure 2C. It is inspired by the redundancy reduction principle described in the work of the neuroscientist H. Barlow.^56^ The two branches of BT process two distorted versions of the same sample to produce their representations. The model measures the cross-correlation matrix between the two learned representations, which is expected to be close to the identity matrix. BT simplifies the training procedure compared with BYOL and SimSiam, which require asymmetric components, such as a predictor layer, as well as operations, including gradient stopping and EMA. A BT model benefits from very high-dimensional output vectors, and its loss function is formulated as

(Equation 9) $$L=\underset{\text{invariance term}}{\underset{︸}{\sum_{i}{\left(1-C_{ii}\right)}^{2}}}+\lambda \underset{\text{redundancy reduction term}}{\underset{︸}{\sum_{i}\sum_{j\neq i}C_{ij}^{2}}},$$

where the cross-correlation matrix computed between the outputs of the two networks along batch direction is defined as

(Equation 10) $$C_{ij}=\frac{\sum_{n}p_{n,i}^{A}p_{n,j}^{B}}{\sqrt{\sum_{n}\left(p_{n,i}^{A}\right)}\sqrt{\sum_{n}\left(p_{n,j}^{B}\right)}},$$

where *n* indexes samples in the batch of size *N*. By minimizing the training objective, the invariance term pushes the diagonal elements of the correlation matrix to 1, which makes the learned representations of the two distorted versions of a sample as close as possible. The redundancy reduction term compresses the correlations between the off-diagonal elements of the correlation matrix. This reduction of the redundancy between output elements in a representation vector results in representations of sufficient disentanglement.

#### Clustering

A general approach to yield pseudo-labels for SSL instead of creating additional data views. Considering that different objects are naturally associated with distinct categories, each category should occupy a separate manifold in the representation space. Deep Cluster,^57^ as shown in Figure 1D, performs two steps iteratively. First, it exploits the K-means clustering method^57^^,^^58^ to group the encoded representations and produce pseudo-labels for each sample. Then, with the created pseudo-labels assigned to each sample, the encoder network can be optimized by minimizing the classification loss, such as by negative log-likelihood function. Instead of the global clustering method of K-means clustering, local aggregation (LA)^59^ allows for modeling more flexible statistical structures by separately identifying neighbors for each example. Moreover, LA proposes an objective function that directly optimizes a local soft-clustering metric, leading to better training efficiency. Another clustering method used in SSL is SwAV,^60^ which introduces online clustering ideas into a Siamese architecture, thus avoiding the time consumption due to the two-step training paradigm. The online clustering assignment provides pseudo-labels within mini-batches, projecting the encoded embeddings to codes (based on the clustering centroids, defined as prototypes). The prototypes are learned along with encoder parameters in a swapped prediction problem. In addition, the authors introduce multi-crop augmentation, enabling the mix of image views of different resolutions.

### Interrelationships between SSL frameworks

The introduced SSL frameworks differ in the way they create the optimization target, i.e., pseudo-labels, for training the model. From the viewpoint of the deformed data's representation, the auto-encoding framework guides it to predict the original data. In this sense, the deformed data can be seen as one view of the sample, while the original data are the other view, which also serves as the pseudo-label for training the auto-encoder. The non-contrastive SSL framework learns the representation of one view by predicting the representation of the other view rather than predicting the other view itself. Suppose the total representation space is limited: contrastive SSL additionally contrasts this representation with some negative samples. This can further restrict the allowed space to represent specific data and can potentially improve the learned representations with better distinctiveness. With multiple views of the same data, non-contrastive and contrastive SSL can directly minimize their distance in representations. Differently, the clustering framework requires no additional-view generation but explicitly groups the learned representations based on the underlying similarity between each input. The clustering centroids are taken as pseudo-labels that attract the learning of the representations of the similar samples. The representations are then centralized to multiple centroids by jointly minimizing the distance of the samples' representations to their centroids.

Recent works have experimentally tested the importance of components for achieving effective SSL models.^61^ The training objectives have been shown to be more important than the network architecture, and the quality of the learned representations can be improved by scaling up the model size and the representation size. Furthermore, the quantity and quality of the negative samples have also shown to be important for the performance of SSL using contrastive learning.^62^

The non-contrastive SSL models, auto-encoding methods, and clustering methods aim at reducing the distance between the latent representations of similar data. Contrastive SSL aims at contrasting the distance between positive samples against the distance to negative ones. The advantages and disadvantages of these two training strategies can be traced back to the difference between generative and discriminative models in the wide field of machine-learning methods. Training SSL without negative samples may be less effective in learning discriminative features between samples but has more potential to code more complete information into representations. Differently, contrastive SSL approaches are expected to learn more discriminative features being compared with negative samples, at expense of dropping common attributes, which are salient to represent the sample itself but are not very informative for distinguishing between samples. Based on this, we infer that non-contrastive SSL may be able to capture representations of more completeness, which should be considered when expecting the SSL model to work as a general feature extractor. However, the parameters of a pre-trained model can be further fine-tuned for a downstream task in a specific domain, a contrastive SSL model can be improved to complement its generalization ability to capture more complete representations, and a non-contrastive SSL model can also be further improved to reduce the redundant information in the representations for the downstream tasks. Even though recent works have conducted comparative experiments of the efficacy of non-contrastive SSL and contrastive SSL,^49^^,^^50^ a clear explanation of their performance differences is still missing.

### Semantic representation for sequential data

To solve a machine-learning problem using sequential data, such as video, text, and audio (including speech signals), the information of different levels in the data should be considered depending on the learning objective. For the tasks that attend to the global information of sequential data, the basic SSL frameworks and methods, introduced in the section SSL frameworks, can be exploited directly on the sequential data, as well as on a transformed, augmented, or segmented version of it. However, since some tasks rely on the transient information of the sequential data, in such a case, the sequential data should be frame-wisely processed to retrieve the semantic features. Each frame, or its representation, e.g., a word embedding for an NLP task, is first taken as an independent sample. Then, a context network is responsible for aggregating the representations along time, yielding a context vector as the representative of the global information of fine temporal resolution. The same principle holds for SSL when processing sequential data. However, attending to the features of different levels affects the way to perform views generation and negatives sampling for SSL training.

#### Views and negatives generation

For the purpose of distilling global information, multiple views of the same data can be created by segmenting sequential data while keeping the temporal coherence and consistency of a signal.^45^^,^^63^ Negative samples of the same form can be generated from other data. Taking video as an example,^45^ in a sequence of image frames, two frames in a short temporal range can be considered as a positive pair, while frames that are far away in the same sequence or from other sequences can be taken as negative samples. To improve the temporal resolution of the representations of sequential data, each frame of the data is taken as an independent sample. Based on this, when performing contrastive SSL, the positive and negative samples can be generated from the frames within the same sequential data. For instance, in order to train a predictive coding model (cf. predictive models), a frame to be predicted is equated to the context vector, which is seen as another view of the frame, while other frames within the same data serve as the negative samples.

#### Predictive models

**Figure 3:**
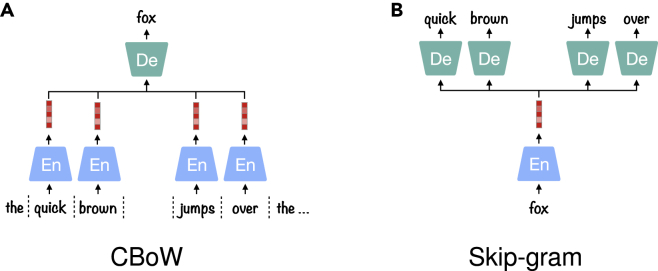
Two architectures of Word2Vec (A) CBoW predicts a single word from the previous and future words. The context words are fed into an En to aggregate a context vector, which is used to produce the target word using a De. (B) Skip gram makes the opposite prediction from CBoW, i.e., predicting previous and future words from a single center word.

The SSL models using auto-encoding framework, introduced in auto-encoding, do not always require predicting the entire original sample, i.e., the prediction can be restricted to only recover the distorted part. This is typically the case for sequential data, as in Word2Vec,^64^ which is used to map one-hot representations of words to word embeddings. In Word2Vec, two formulations are used to learn underlying word representations: Continuous Bag-of Words (CBoW) and Skip-gram, depicted in Figures 3A and 3B, respectively. CBoW is trained to predict a single word from its context words, whereas Skip-gram does the opposite, aiming at predicting the left and right context words of a single input word. CBoW performs better in learning syntactic relationships between words; however, it is prone to overfit frequent words. Differently, Skip-grams are better at capturing semantic relationships and suffer less from overfitting, leading to a more effective solution in learning representations for general purposes.^65^ The success of Word2Vec is based on the consistency of the context surrounding the component to predict.

**Figure 4:**
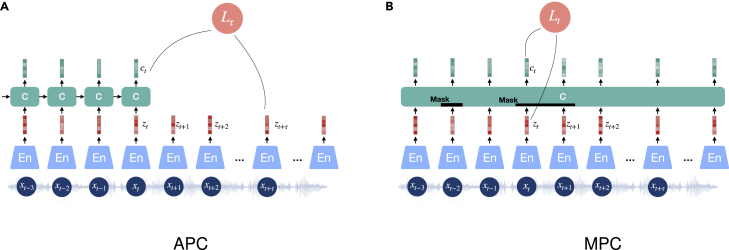
Diagrams of auto-regressive predictive coding (APC) and masked predictive coding (MPC) To apply contrastive loss, the embeddings $z_{n}$, except for the one to predict, can be taken as negatives (distractors). APC shown in (A); MPC shown in (B).

Predictive coding is suggested to be used for tasks concerning transient information in sequential data, such as NLP and some speech-based tasks. An auto-regressive model can learn representations by making predictions of future information conditioning on past context. APC^66^ encodes segments of sequential data into representations (cf. Figure 4A). An additional context network aggregates these representations up to the current time step. Hence, the context network is usually a recurrent neural network (RNN) for modeling the temporal information. Its output context vector is then used to predict the next audio representations, for example, τ steps ahead of the current time step. The APC method codes only on a sequence in the forward direction. In order to achieve a representation conditioned on both directions (past and future), a combination of separately trained models for forward and backward directions is needed. Alternatively, a bidirectional architecture can be realized using MPC, presented in bidirectional encoder representations from transformers (BERT),^67^ which masks parts of the input signals that are subsequently predicted by conditioning on the context from both directions (cf. Figure 4B). Transformer encoders and bidirectional RNNs have been considered as context networks for realizing MPC. The MPC approaches can learn effective representations of sequential data in a non-auto-regressive way and hence achieve considerable speed up in training. Besides, such models seem to be very similar to a masked auto-encoder.^68^ Similarly, a specific non-auto-regressive predictive coding (NPC)^69^ has been recently proposed, which also applies a mask on its model input but learns representations based on local dependencies of an input sequence rather than globally.

**Figure 5:**
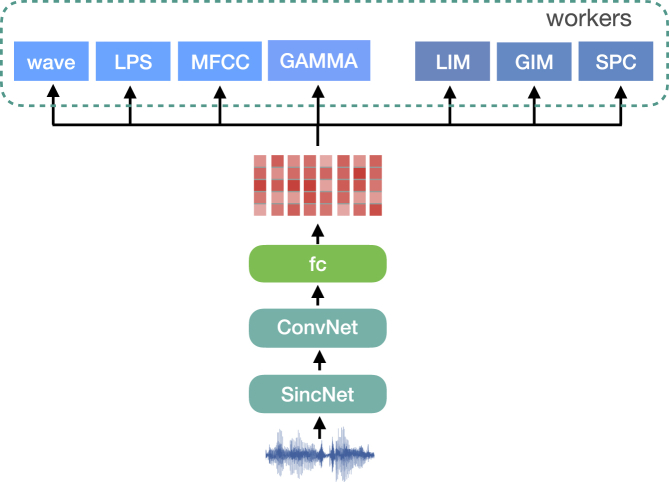
Diagram of PASE

The optimization of Word2Vec, APC, and MPC models can be performed by minimizing the prediction errors or in a contrastive learning way.^42^^,^^70^ Specifically, contrastive predictive coding (CPC)^42^ can exploit an APC architecture optimized to predict the correct future information based on the aggregated global context from past frames. In addition, to maximize the similarity between the context vector $c_{t}$, serving as the pseudo-label, and future audio representation $z_{t+\tau }$, CPC gets use of negative samples to improve the representation discrimination in training objectives such as InfoNCE loss (cf. Equation 4):

(Equation 11) $$L\left(c_{t},z_{t+\tau },z_{n\in \left[1\text{,}N-1\right]}^{-}\right)=E\left[-\text{log}\frac{e^{c_{t}^{\text{T}}z_{t+\tau }}}{e^{c_{t}^{\text{T}}z_{t+\tau }}+\sum_{n=1}^{N-1}e^{c_{t}^{\text{T}}z_{n}^{-}}}\right],$$

where $z_{n}^{-}$ denotes a negative data point sampled from the proposal distribution of $z_{t+\tau }$, i.e., randomly sampled from the sequence *z*. From the perspective of the loss construction, taking the future frame to be predicted as the anchor, the context vector containing global information from the past is seen as the positive, while the other frames are considered negatives. In CPC v.2,^71^ the model is scaled up to achieve larger model capacity, and the batch normalization is replaced by layer normalization, as batch normalization is found to harm downstream tasks for CPC frameworks.^71^ Moreover, patch-based augmentation is introduced in order to add more diversity to the model’s input.

Similarly, for MPC, the contrastive training objective can be formalized as

(Equation 12) $$L\left(c_{t},z_{t},z_{n\in \left[1\text{,}N-1\right]}^{-}\right)=E\left[-\text{log}\frac{e^{c_{t}^{\text{T}}z_{t}}}{e^{c_{t}^{\text{T}}z_{t}}+\sum_{n=1}^{N-1}e^{c_{t}^{\text{T}}z_{n}^{-}}}\right].$$

However, MPC, widely known as a powerful replacement of other predictive coding models used to process sequential data such as APC, is essentially presented and works in the same way as the auto-encoding framework. The approach has also been adopted for processing speech signal, e.g., wav2vec 2.0,^72^ which will be introduced in detail in the following section.

## Audio SSL

Depending on whether transient information is essential on a given audio task, different model frameworks and training objectives should be selected for audio SSL. The basic SSL frameworks (cf. SSL frameworks) aim to train a model to encode the global information of its input signal into a representation vector. Since the learned representation is void of temporal resolution, such approaches are suitable for non-speech audio applications, such as acoustic scene classification (ASC), whose audio recordings own good consistence in signal. Contrastive SSL and the Siamese network are mostly exploited for this learning purpose. For this, the positive pair can be segmented from the same audio recording, or its spectrogram or Mel representations, and the negative pair can be extracted from different recordings.^73^^,^^74^^,^^75^ Besides, the model learned though these approaches can be fitted to solve paralinguistic downstream tasks, owing to the relatively slower change of the non-semantic aspects of the speech signal compared with its phonetic and lexical aspects.^76^

To capture the transient information in speech for applications requiring semantic features, representation learning should be performed on each short-term segment of the speech signal or each frame of its time-frequency representation. To this end, predictive coding like APC and MPC can be used to learn a representation for each time step.^42^^,^^72^^,^^77^^,^^78^^,^^79^^,^^80^ These short-term segments or the representation frames can be treated as independent samples for SSL training due to the fast time variance in a speech signal, enabling contrastive training of these predictive coding models using signal segments within the same speech sequence.

In the following, we categorize the approaches for audio SSL based on the above considerations. A summary table of typical audio SSL methods is shown in Table 2. It is worth mentioning that several artificial intelligence (AI) communities, including Hugging Face (https://huggingface.co/) and Fairseq (https://github.com/facebookresearch/fairseq), keep updating their open-source toolkits to promote the development and use of audio SSL methods.

**Table 2:** An overview of the recent audio self-supervised learning methods

Model	Speech	Input format	Framework	Encoder	Loss	Inspired by
LIM^73^ (2019)	✓	raw waveform	1(b)	SincNet	BCE, MINE, or NCE loss	SimCLR
COLA^74^ (2021)	✗	log mel-filterbanks	1(b)	EfficientNet	InfoNCE loss	SimCLR
CLAR^81^ (2021, semi)	✗	raw waveform log mel-spectrogram	1(b)	1D ResNet-18 ResNet-18	NT-Xent + cross-entropy	SimCLR
Fonseca et al.^75^ (2021)	✗	log mel-spectrogram	1(b)	ResNet, VGG, CRNN	NT-Xent loss	SimCLR
Wang et al.^82^ (2020)	✗	raw waveform + log mel-filterbanks	1(b)	CNN ResNet	NT-Xent loss + cross-entropy	SimCLR
BYOL-A^83^ (2021)	✗	log mel-spectrogram	2(a)	CNN	MSE loss	BYOL
Carr^37^ (2021)	✓	MFCCs	1(a)	context-free network	Fenchel-Young loss	–
Ryan^38^ (2020)	✗	constant-Q transform spectrogram	1(a)	AlexNet	triplet loss	–
Speech2Vec^90^ (2018)	✓	mel spectrogram	3	RNN	MSE loss	Word2Vec
Audio2Vec^89^ (2020)	✓✗	MFCCs	3	CNN	MSE loss	Word2Vec
DeCoAR^91^ (2020)	✓	log filterbank features	3	RNN	L1 loss	Word2Vec
Audio Word2Vec^195^ (2019)	✓	MFCCs	3	RNN	MSE loss	Word2Vec
Mockingjay^95^ (2020)	✓	mel spectrogram	4(b)	transformer	L1 loss	BERT
TERA^96^ (2021)	✓	log mel spectrogram	4(b)	transformer	L1 loss	BERT
Audio ALBERT^98^ (2021)	✓	log mel spectrogram	4(b)	transformer	L1 loss	BERT
DAPC^99^ (2021)	✓	spectrogram	4(b)	transformer	modified MSE loss + orthogonality penalty	BERT
PASE^85^ (2019)	✓	raw waveform	1(a)	SincNet + CNN	L1, BCE loss	MTL
PASE+^87^ (2020)	✓	raw waveform	1(a)	SincNet + CNN + QRNN	MSE, BCE loss	MTL
APC^66^ (2019)	✓	log mel spectrogram	4(a)	RNN	L1 loss	–
VQ-APC^114^ (2020)	✓	log mel spectrogram	4(a)	RNN, transformer	L1 loss	–
NPC^69^ (2021)	✓	log mel spectrogram	–	CNN + masked CNN	L1 loss	–
CPC^42^ (2018)	✓	raw waveform	4(a)	ResNet + GRU	InfoNCE loss	–
CPC v2^71^ (2020)	✓	raw waveform	4(a)	ResNet + masked CNN	InfoNCE loss	–
CPC2^93^ (2021)	✓	raw waveform	4(a)	ResNet + LSTM	InfoNCE loss	–
wav2vec^77^ (2019)	✓	raw waveform	4(a)	1D CNN	contrastive loss	–
VQ-wav2vec^78^ (2019)	✓	raw waveform	4(a)	1D CNN + BERT	contrastive loss	BERT
wav2vec 2.0^72^ (2020)	✓	raw waveform	4(b)	1D CNN + transformer	contrastive loss	BERT
HuBERT^112^ (2021)	✓	raw waveform	4(b)	1D CNN + transformer	contrastive loss	BERT
WavLM^113^ (2022)	✓	raw waveform	4(b)	1D CNN + transformer	contrastive loss	BERT

Model, speech (i.e., whether a method addresses speech tasks or it is designed for general audio representations), framework (referring to Figures 1, 2, 3, and 4), encoder, loss, and the previous technology by which the methods are inspired, are given.

### General-purpose audio SSL

#### Contrastive SSL

The methods differ in the used audio input formats, such as LIM,^73^ COLA,^74^ CLAR,^81^ and the work by Fonseca et al.,^75^ and expand the SimCLR approach for learning auditory representations. The LIM model,^73^ which aims at learning useful speaker representations, directly processes speech samples with the expectation of maximizing local mutual information between the encoded representations of chunks of speech sampled from the same utterance. In COLA^74^ and the work by Fonseca et al.,^75^ the presented models take segments randomly extracted from time-frequency features along the temporal direction. Several data augmentations are adopted for the patches before feeding to the model in Fonseca et al.^75^ such as random size cropping and Gaussian noise addition, as well as their proposed mix back, i.e., mixing the incoming patch with a background patch but ensuring that the incoming patch is dominant in the mixture. In CLAR,^81^ the paired views of the model’s input are generated by applying data augmentations on raw audio signals and time-frequency audio features, for which effective compositions of data augmentation are explored. In addition, the authors suggest combining the training of SSL with supervised learning using substantially less labeled data than a complete supervised-learning setup. Correspondingly, the contrastive loss and cross-entropy loss are added together as the complete training objective. This method provides significant improvements in terms of convergence speed and representation effectiveness, with respect to using SSL only. Similarly, Wang^82^ also suggests training audio SSL models with different formats of an audio sample. More precisely, the training objective is to maximize the agreement between the raw waveform and its spectral representation. The approach is shown to be effective for downstream classification tasks on both AudioSet and ESC-50 datasets.

#### Siamese network

BYOL, as a representative SSL framework using the Siamese network, has been adopted in the audio domain, named BYOL-A,^83^ which learns representations from a single audio without using negative samples. To generate two views of an audio segment as the input of the Siamese network, its log mel spectrogram is unequally processed using two data augmentation techniques, i.e., Mixup and Random Resize Crop (RRC), which randomly resizes and crops the signal in the two model branches. Pre-normalization and post-normalization are additionally applied to stabilize the data-augmentation procedure. The remaining parts of the model are consistent with the architecture of BYOL. The method has been found to be effective for learning a general-purpose audio representation for several classification tasks, including those based on non-speech signals, e.g., music instrument family classification, as well as speech signals, e.g., speaker identification, language identification, and speech command recognition.

#### Auto-encoding

Another predictive model, i.e., Audio Word2Vec,^37^ makes use of a sequence-to-sequence auto-encoder to represent audio frames into latent attributes. For this, two RNNs serve as the encoder and decoder, which are jointly optimized by minimizing the reconstruction error. Meanwhile, a segmentation gate is introduced in Audio Word2Vec to estimate word boundaries, enabling it to segment an utterance into its spoken words. The parameters of the segmentation gate can be updated based on rewards computed using reinforcement learning. The auto-encoder and the segmentation gate are updated in an iterative way, i.e., optimizing the parameters of one of them while fixing the other one.

As a special case of applying auto-encoding, Carr et al.^37^ proposed a training strategy based on permutations, i.e., training a model that can reorder shuffled patches of an audio spectrogram, analogous to solving a jigsaw puzzle.^34^ The method draws inspiration from “Shuffle and Learn” ^84^ and has also been considered in another work for industrial audio classification.^38^ In Carr et al.,^37^ the authors also leverage differentiable ranking to integrate permutation inversions into an end-to-end training, which enables solving the permutation inversion for the whole set of permutations, i.e., reducing the space of permutations that might be exploited and performing the reordering as a classification task.

### Semantic representation for speech

#### Multi-task resembling

The first to be introduced is the problem-agnostic speech encoder (PASE; Figure 5),^85^ an approach that combines a convolutional neural network (CNN) encoder with multiple neural decoders, defined as workers in the literature. The workers, fed with learnt representations from the encoder, aim at solving regression or binary discrimination tasks. The regression tasks include, for instance, recovering the raw audio waveform in a similar way as auto-encoding, the log power spectrogram, mel frequency cepstral coefficients (MFCCs), and prosody. Regarding binary discrimination tasks, contrastive training objectives are used. Local info max (LIM) exploits the same method as in Ravanelli and Bengio^73^ to maximize local information for the embedding of each frame created by the PASE encoder. By averaging the embeddings of these frames, maximize global information (GIM) is also considered to be complementary to the LIM. Moreover, the idea of CPC^42^ is also adopted with changes made in sampling positive and negative samples. Each self-supervised task is expected to provide a different view of the speech signal; jointly solving self-supervised problems pushes the views into a unique representation that contains meaningful speech information such as speaker voice print, phonemes, or emotions. In addition, to process the raw waveform as the encoder input, the SincNet^86^ model is used as the first stage of PASE, which performs a convolution with a set of parametrized Sinc functions that implement rectangular band-pass filters. PASE+^87^ incorporates additional data-augmentation techniques and more effective workers. The CNN encoder is combined with a quasi-RNN (QRNN)^88^ for capturing long-term dependencies in sequential data in a more efficient way.

#### Word2Vec

Other typical works include Audio2Vec,^89^ Speech2Vec,^90^ and DeCoAR,^91^ inspired by Word2Vec,^64^ as introduced in predictive models. The former two works learn audio representations using CBoW and skip-gram formulations, while the last only considers a method similar to CBoW. In the CBoW formulation, the task is to reconstruct a temporal spectrogram slice of pre-determined duration from a number of past and future slices. The method has also been shown to be effective for ASC in Gontier et al.^92^ Differently, the roles of the target and surrounding slices are reversed in the skip-gram formulation. Audio2Vec and Speech2Vec mainly differ in the following aspects: (1) Speech2Vec applies audio segmentation, by using an explicit forced alignment technique, in order to isolate audio slices corresponding to each word. The forced alignment segmentation may introduce supervision to some extent. (2) Audio2Vec requires no explicit assistance and hence completely removes the need for supervision. (3) Unlike neural network architectures, Speech2Vec is built based on an RNN encoder-decoder, and Audio2Vec is built of stacks of CNN blocks. (4) As model input, Speech2Vec processes the mel spectrogram of an audio, while Audio2Vec operates on MFCCs. (5) In Audio2Vec, the TemporalGap formulation is additionally introduced, which requests that the model estimates the absolute time distance between two (randomly sampled) slices taken from the same audio clip.

#### CPC

Van den Oord proposed CPC,^42^ which can effectively learn representations by predicting the future in a latent space using an auto-regressive model, showing very promising results for audio, images, text processing, and reinforcement learning. For audio, a strided convolutional network is used to encode raw audio to its latent representation. Then, a gated recurrent unit (GRU)-RNN aggregates the information from all the past time steps to form a context vector. More importantly, contrastive loss is applied to learn more discriminative representations by contrasting the true future to noise representations, given an aggregated context vector. Speech signals can be pre-processed by using a time-domain data augmentation library, such as WavAugment,^93^ in order to achieve more powerful representations by CPC. The library contains several data augmentation (DA) techniques, including pitch modification, additive noise, reverberation, band reject filtering, or time masking, to name a few. In Kharitonov et al.,^93^ the authors define a CPC2 model, which replaces the GRU-RNN of CPC by a two-layer long short-term memory (LSTM)-RNN and replaces the linear prediction network by a single multi-head transformer layer, leading to better training efficiency without harming representation performance.

**Figure 6:**
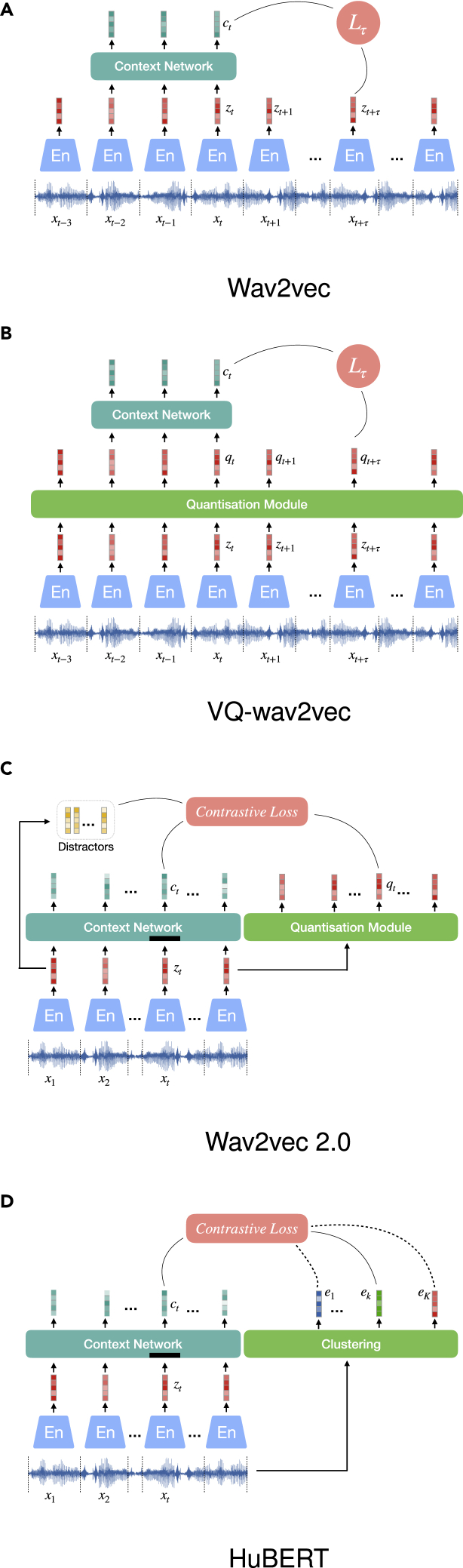
Wav2vec series and HuBERT

Wav2vec,^77^ as shown in Figure 6A, adjusts the CPC structure to a fully convolutional architecture, enabling easy parallelization over time on hardware. One CNN encodes the raw waveform into audio representations for each time step, and the other captures global context information into a context vector. Specifically, the wav2vec approach is optimized by minimizing contrastive loss for each step k=1,…,K:

(Equation 13) $$L_{k}=-\sum_{i=1}^{T-k}(\text{log}\sigma \left(z_{i+k}^{\text{T}}h_{k}\left(c_{i}\right)+\lambda E[\text{log}\sigma \left(-{\tilde{z}}^{\text{T}}h_{k}\left(c_{i}\right)\right]\right),$$

where $\tilde{z}$ is the distractor uniformly sampled from the audio representations, $\sigma \left(x\right)=1/\left(1+e^{-x}\right)$. $\sigma (z_{i+k}^{\text{T}}h_{k}\left(c_{i}\right)$ stands for the probability of $z_{i+k}$ being the true future sample of $c_{i}$, and $h_{k}\left(c_{i}\right)=W_{k}c_{i}+b_{k}$ applies an affine transformation to $c_{i}$, similar as in Oord et al.^42^ The total loss sums up considering *K* steps; $L=\sum_{k=1}^{K}L_{k}$ is minimized for training. After pre-training, the affine projection layer is removed for creating the learned representations from the raw audio. This method moves beyond phoneme-based automatic speech recognition (ASR), as explored in Oord et al.,^42^ and substantially improves a character-based ASR system.

A follow-up work by Baevski et al.^78^^,^^94^ (cf. Figure 6B) implements a vector quantization module after the wav2vec encoder in order to discretize the audio representations. This aims to find, for each representation, the closest embedding and codeword from a fixed size codebook. The discrete representations are fed into the context network and then optimized in the same way as for wav2vec. We will introduce the principle of vector quantization in detail, comparing its two realization methods, i.e., K-means clustering and Gumbel-Softmax, in the section representation quantization.

#### MPC

Masked acoustic model (MAM) (cf. Figure 4B), built on transformer architecture, masks some parts of an audio input and reconstructs the entire original input in order to fill the masked parts that are not known by MAM during training.^95^^,^^96^ Such model can be optimized by minimizing the reconstruction error, contrastive loss, and clustering.

##### Optimization with reconstruction error

**Figure 7:**
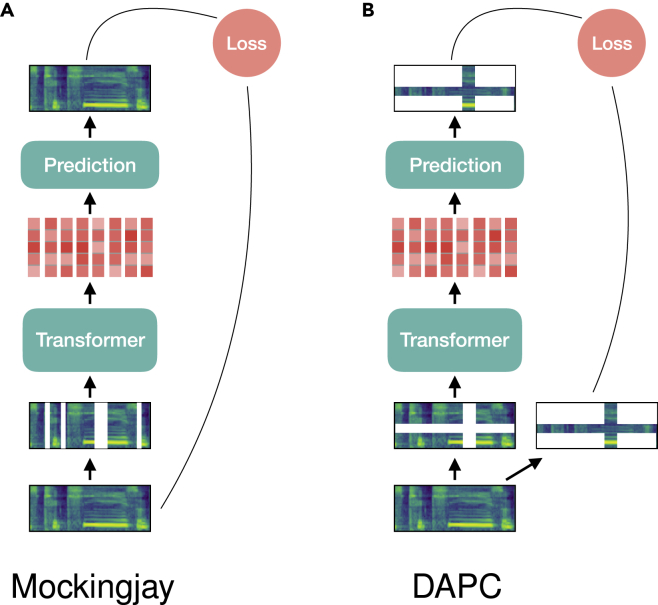
MPC models for audio SSL

Mockingjay^95^ (cf. Figure 7A) takes the mel spectrogram as input acoustic features and exploits transformers to code randomly masked frames into audio representations. The encoded representations are mapped to predict the complete frames using a projection head built of two-layer MLP with layer normalization. The transformer encoder and projection head are jointly optimized by minimizing the L1 reconstruction loss. The effectiveness of self-attention in transformer encoders has been further explored in Yang et al.;^97^ the authors also provide a visualization tool for understanding the attention, based on which several attention refinement techniques are proposed to improve model performance. Audio ALBERT^98^ has the same network architecture as Mockingjay, but the parameters are shared across all its transformer encoder layers, thus achieving a faster inference and increasing training speed without harming the performance of two evaluation downstream tasks, i.e., speaker classification and phoneme classification. In transformer encoder representations from alternation (TERA),^96^ the authors extend the used masking procedures, including replacing contiguous segments with randomness, masking along the channel axis, and applying Gaussian noise for pre-training the transformers. This resulted in a better representation performance than the one shown by Mockingjay and audio ALBERT for downstream tasks, phoneme classification, keyword spotting, and speaker classification.^96^ In addition, it shows also promising results for ASR tasks based on the Librispeech and TIMIT datasets.

Unlike the works that predict the entire audio frames from their masked version, DAPC^99^ (cf. Figure 7B) proposes a method to only predict the missing components along the time and frequency axes of an audio spectrogram by minimizing a masked reconstruction loss. The method is also regarded as an extension of CBoW, for which the input’s masked spectrogram can be easily generated using SpecAugment,^100^ and hence, the missing parts to be predicted are not only temporal frames but are also frequency bins.

##### Optimization using contrastive loss

Wav2vec 2.0 Figure 6D exploits a MAM as in the section MPC but is optimized using a contrastive loss, i.e., InfoNCE.^42^ The raw audio is encoded using multiple one-dimensional (1D)-CNN layers, and the resulting representations are partly masked before being sent to a transformer network to learn contextualized representations. The networks are jointly trained to contrast the true representations from distractors given the contextualized representations. Similar to VQ-wav2vec, wav2vec 2.0 applies product quantization, too; however, the quantized vector $q_{t}$ for each time step is not fed into a context network but is only used in the objective function:

(Equation 14) $$L=E\left[-\text{log}\frac{e^{c_{t}^{\text{T}}q_{t}/\tau }}{\sum_{\tilde{q}∼Q_{t}}e^{c_{t}^{\text{T}}\tilde{q}/\tau }}\right],$$

where $\tilde{q}∼Q_{t}$ includes $q_{t}$ and *K* distractors. In addition to this InfoNCE, the training loss is regularized by a diversity loss $L_{d}$ to encourage the model to make better use of the codebook, which is detailed in wav2vec 2.0 and shows very promising results for ASR tasks evaluating on both Librispeech^101^ and TIMIT^102^ datasets.

Wav2vec 2.0 has been further investigated for analyzing its efficacy in terms of cross-domain shift^103^ and cross-language.^104^^,^^105^ To explore the effect of cross-domain shift, the data for pre-training, fine-tuning, and evaluation are from different domains. The authors conclude that the matching conditions between data of pre-training and testing are very important in order to achieve satisfying speech recognition results. Moreover, pre-training on multiple domains can improve the generalization ability of the learned representations. The task of learning multi-lingual speech representations has also been undertaken based on wav2vec 2.0^104^ and Babu et al.,^105^ as well as by a bidirectional CPC model in Kawakami et al.^106^ Besides, the architecture of wav2vec 2.0 also reveals promising improvements in learning general-purpose audio representations for non-speech audio tasks as shown in Srivastava et al.,^107^ where the transformer is replaced by a conformer.^108^

The use of a codebook in wav2vec 2.0 aims to restrict the number of possible audio representations, emulating the situation in the NLP domain, i.e., finite words exist, and each of them has a unique embedding. However, the situation is different for real-world noisy audio due to different recording environments. To solve some difficulties in utilizing a codebook observed for wav2vec 2.0, Sadhu et al.^109^ proposed wav2vec-C, which uses an explicit consistency network to reconstruct the original input features from the encoded discrete representations by wav2vec 2.0. Hence, it can be seen as integrating wav2vec 2.0 and the vector-quantized variational auto-encoder (VQ-VAE)^110^ in a single model. The reconstruction error is added to the InfoNCE loss of wav2vec 2.0, providing a regularization effect in learning the speech representation and enforcing it to explicitly carry essential information for recovering the input features. This method has yielded some additional improvement in ASR on real-world far-field noisy data compared with the original wav2vec 2.0.^72^

Recently, data2vec^111^ unified the same SSL regime that works for other different modalities, including vision and language in addition to speech. Using front-end modality-specific encoding modules for different data types, a standard transformer is then trained in order to predict representations of the entire input data given the partially masked input.

##### Optimization using clustering

Differently, hidden-unit BERT (HuBERT)^112^ trains a MAM model without contrastive learning and avoids using vector quantization. Instead, inspired by DeepCluster,^57^ the learned audio representation is paired with a pseudo-label provided by performing clustering, such as K-means, to the MFCCs of the input audio. To train this model, the losses for masked and unmasked frames are formulated identically as a contrastive loss:

(Equation 15) $$L=E\left[-\text{log}\frac{e^{{\left(Ac_{t}\right)}^{\text{T}}e_{k}/\tau }}{\sum_{k=1}^{K}e^{{\left(Ac_{t}\right)}^{\text{T}}e_{k}/\tau }}\right],$$

where *A* is a matrix to project the context vector $c_{t}$, which is trained to be close to the centroid of its belonging cluster $e_{k}$ and away from other centroids. The losses of masked and unmasked frames are added together to be minimized. Two operations can be additionally performed on the clustering to improve the representation quality. First, the method benefits from cluster ensembles, as the K-means clustering can be of different numbers of clustering centers, creating targets of different granularity. Second, the pseudo-labels can be refined throughout the learning process by applying clustering to the learned audio representation, which is expected to be of better quality over MFCCs.

Later, WavLM^113^ improves the model robustness of the same network as HuBERT by augmenting some training data with additive noise (including overlapping speech) and subsequently trains the model with the pseudo-labels created from the original clean data (in the masked region) using a clustering method. By doing this, the trained SSL model can learn representations of higher robustness against noise when performing speech tasks. This also enhances the model’s capability to process more complex audio scenarios, enabling it to learn representations for non-speech downstream tasks.

### Representation quantization

With the aim to emulate a written language having a finite vocabulary of discrete units, i.e., words or sub-words, vector quantization can convert a speech representation in the continuous space into the discrete space of finite possible representations. The idea is used in VQ-wav2vec, wav2vec 2.0, and HuBERT, as previously introduced, as well as other methods including VQ-VAE,^110^ VQ-APC,^114^ NPC,^69^ and SeqRA-AE.^80^ Specifically, the quantization aims to find each latent feature $z_{t}$, i.e., speech representation at time step *t*, and the closest embedding from a fixed size codebook $e\in R^{V\times d}$ containing *V* codewords of size *d*. This can be realized by using either the Gumbel-Softmax approach or K-means clustering.

#### Gumbel-Softmax

**Figure 8:**
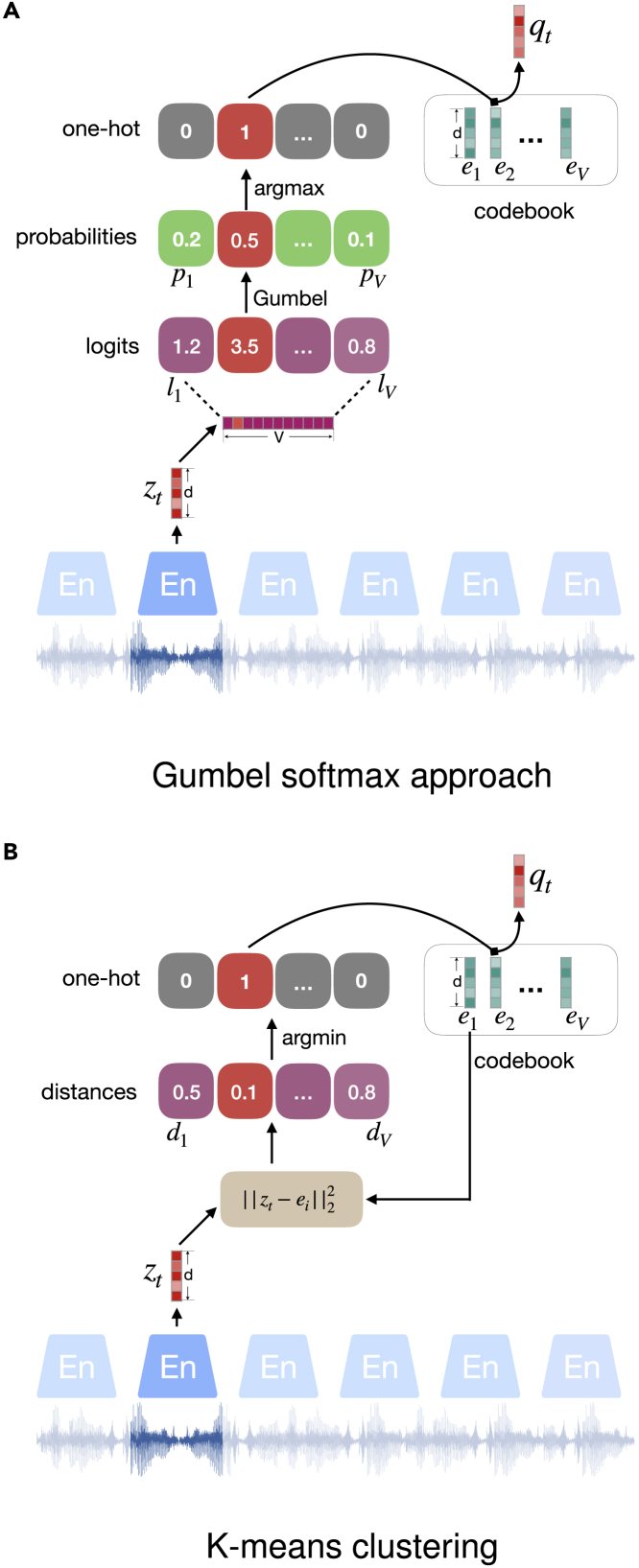
Diagrams illustrating the mechanisms to select codewords from a codebook

As depicted in Figure 8A, the first method selects a codeword by learning a one-hot vector from the speech representation. Hence, the length of the one-hot vector equals the possible number of codewords, and the codeword is of the same size of the speech representation. To this end, the speech representation is projected onto a vector of length *V* via two dense layers. The resulting logits $l_{1,\ldots ,V}$ are fed into Gumbel-Softmax to emit the probabilities of selecting each codeword:

(Equation 16) $$p_{v}=\frac{e^{\left(l_{v}+n_{v}\right)/\tau }}{\sum_{k=1}^{V}e^{\left(l_{k}+n_{k}\right)/\tau }},$$

where n=−log(−log(u)) stands for Gumbel noise, in which the *u* is sampled from the uniform distribution U(0,1), and τ is a non-negative temperature parameter. Gumbel-Softmax is a differentiable approximation of argmax, specifically when τ approaches 0, Equation 16 becomes the same as argmax. Hence, the output of Gumbel-Softmax approximates a one-hot vector. In practical training, argmax is further used to turn the possibilities into the one-hot vector, with the “1” indicating the index of the codeword to choose. However, the back propagation only computes the gradients with respect to Gumbel-Softmax outputs—the exact probabilities rather than the one-hot result—for the parameters’ optimization.

Using a single codebook for coding representations tends to mode collapse, i.e., only some of the codewords are actually used. To solve this issue, multiple codebooks are used for quantization.^115^ To use *G* codebooks, a speech representation should be split into G isometric representation segments, each of size d/G. Then, a codeword from one codebook can be chosen in the same way as introduced above. The complete quantized representation concatenates these chosen codewords into one vector.

To encourage the use of *V* codebook entries equally often, a diversity loss can be additionally used, as shown, e.g., in wav2vec2.0. The diversity loss for *G* codebooks is minimized to maximize the entropy of the probabilities:

(Equation 17) $$L_{\text{d}}=\frac{1}{GV}\sum_{g=1}^{G}-H\left({\bar{p}}_{g}\right)=\frac{1}{GV}\sum_{g=1}^{G}\sum_{v=1}^{V}\;{\bar{p}}_{g,v}\text{log}\;{\bar{p}}_{g,v},$$

expecting to make use of all possible codewords with the same frequency.

An application to utilize the quantization results is to group quantized audio representations into phoneme sequences, which is named phonetic clustering in SeqRA-AE.^80^ In this work, the discrete representation is learned in an auto-encoder architecture with vector quantization. Moreover, the consecutive repeated quantized representations are further grouped to form phonetic units. Each phoneme can therefore correspond to several repeated codewords, which is similar to the format of connectionist temporal classification (CTC).^116^

#### K-means clustering

As an alternative solution to realize differentiable vector quantization, K-means clustering can assign a speech representation to a cluster according to the distance between the representation and the clustering centroid. As shown in Figure 8B, the distances between $z_{t}$ and all the centroids are calculated; then, the closet centroid is selected as the quantized representation. To train a model that learns discrete speech representations using this approach, additional loss components are needed:

(Equation 18) $$L_{cluster}=\left({\left\|\text{sg}\left(z\right)-q\right\|}^{2}+\gamma {\left\|z-\text{sg}\left(q\right)\right\|}^{2}\right),$$

where sg(.) is the stop gradient operator and γ is a hyper-parameter. By immunizing the loss, the term $||\text{sg}\left(z\right)-q|{|}^{2}$ freezes the encoder output *z* and forces the codewords *Q* to be closer to the encoder output. The other term $||z-\text{sg}\left(q\right)|{|}^{2}$ drives each encoder output to be close to one codeword, which is one centroid of the K-means clustering. Note that for HuBERT, K-means clustering is similarly used to create pseudo-labels. However, the optimization of the learned speech representation is trained using all cluster centroids in a contrastive way, i.e., contrast its similarity to the belonging centroid with the similarity to other centroids, as formulated in Equation 15.

### Regression tasks

Although the research of audio SSL began with learning representations able to solve classification downstream tasks, the application of SSL to audio regression tasks has recently received attention. An example can be seen in the popular benchmark SUPERB-SG,^117^ which extends its previous version SUPERB^118^ containing only classification tasks with five audio regression tasks, such as speech enhancement and separation or voice conversion, among others.

In fact, we noticed that the classic formulation of several front-end audio processing tasks that have been explored for a long history are essentially using the framework audio SSL, especially auto-encoding predictive models as the one shown in Figure 1A. Indeed, methods for speech enhancement (SE), i.e., able to process a noisy audio input and output clean speech, has been presented.^119^^,^^120^ For generating the noisy input, clean speech is typically mixed with a noise recording. This is exactly the same as the processing of input to an auto-encoding predictive model, while the noise addition is seen as a step for DA. Hence, the latent features in the middle layers of an SE model are seen as a kind of audio representation of the clean speech. The formulation is not limited to SE but is applicable to all the pre-processing tasks that aim at predicting an audio of interest from additive and multiplicative noise or interference, such as source separation, de-reverberation, and echo cancellation.

In some very recent works, audio SSL approaches have been chosen to solve some special regression tasks related to SE^121^^,^^122^^,^^123^ and source separation.^124^ In Wang et al.,^121^ a pair of VAEs, named clean auto-encoder (CAE) and mixture auto-encoder (MAE), were exploited. A CAE is trained to learn representations of clean speech by minimizing the reconstruction error of its input spectrogram. An MAE encodes a noisy utterance and enforces the encoded representation into the same latent space of the CAE by using a cycle-consistency loss term. This paradigm leans feature mapping or spectral mapping from the domain of mixtures to the domain of clean sounds. Mixture invariant training (MixIT) is proposed in Wisdom et al.^125^ for solving unsupervised sound separation. In MixIT, a separation network takes a mixture of multiple single-channel acoustic mixtures (MOMs) as model input, where each of the acoustic mixtures is comprised of several speech sources. The separation network decomposes the MOMs into separate audio sources, which are then selected to be re-mixed up to approximately each acoustic mixture of the MOM. Similar to permutation invariant training (PIT),^126^ the remix matrix is optimized by choosing the best match between the separated sources and the acoustic mixtures. The method shows improvements for reverberant speech separation and universal sound separation and is effective for SE, too. MixIT, as a typical universal sound-separation method, is verified to be a valid DA approach to generate positive views for contrastive learning.^127^ It learns to associate sound mixtures with separated channels, thus this retaining semantic structure in learned representations. Finally, using denoising pre-training is an alternative solution to solve the permutation switching problem of source separation.^124^ In this work, the authors use speech denoising as a self-supervised pre-training task to learn the structure information of speech from large-scale data. The model is subsequently fine-tuned with the normal training objective of source separation. As knowledge about the speech structure has been captured in the pre-trained model, it relaxes the permutation problem.

To develop an SE system specialized in a particular person (PSE), Sivaraman and Kim^122^ present two SSL algorithms, pseudoSE and contrastive mixtures (CMs), for extracting speaker-specific discriminative features. A pseudoSE model is trained to recover a pre-mixture signal (i.e., clean speech contaminated by noise) from a pseudo-source (i.e., a mixup of the pre-mixture signal and additional noise). The CM method generalizes the training via contrastive learning, for which a positive pair shares the same pre-mixture signal (but is deformed with different additional noises), while a negative pair stems from two different pre-mixture sources mixed with the same additional noise. The trained model, using either contrastive or non-contrastive SSL, is trained to recover pre-mixture sources rather than clean speech, and hence, it requires fine-tuning for the downstream task. Data purification (DP)^128^ is later introduced in the pseudoSE training. Specifically, a separate model is trained to estimate the segmental signal-to-noise ratio (SNR) of the pre-mixture signals, measuring the different importances of the audio frames. Injecting the importance measurement in pseudoSE training enables the model to benefit from segments of higher quality, and hence, enables it to derive more meaningful speaker-specific features.

### Multi-modal audio representation

The successful adoption of SSL has spread over many academic and industrial fields, including, but not limited to, CV, audio processing, and NLP, to cite a few. Moreover, exploiting multi-modal SSL has also been explored for a variety of applications whereby the representation learning of different modalities can be performed simultaneously. The mutual complementarity between different modalities, treated as different views representing one unique object, is beneficial for the representation learning of each considered modality. In this section, we discuss multi-modal SSL approaches that use audio as one modality. Most of these works are based on audio-visual processing, which aims, for example, at determining the correspondence between video frames and its audio sequence. Other visual-audio methods, similar to the SSL works previously described, make use of the synchronization of the visual and audio streams of a video, taking the two views as input of a Siamese network. In this case, each modality of the two can be seen as the supervisory signal for the other. Audio representation can also be learned during a task of video generation, where the representation of each segment of an utterance is expected to carry adequate speech information in order to transfer the knowledge into video frames. Some more interesting approaches are motivated by classic tasks in CV, i.e., object segmentation and localization, and audio processing, i.e., source separation. On the other hand, text is also considered as one modality that assists in the learning of speech representations, because speech and text have a similar linguistic structure.

### Audio and visual

#### Visual-audio correspondence (AVC) decision

**Figure 9:**
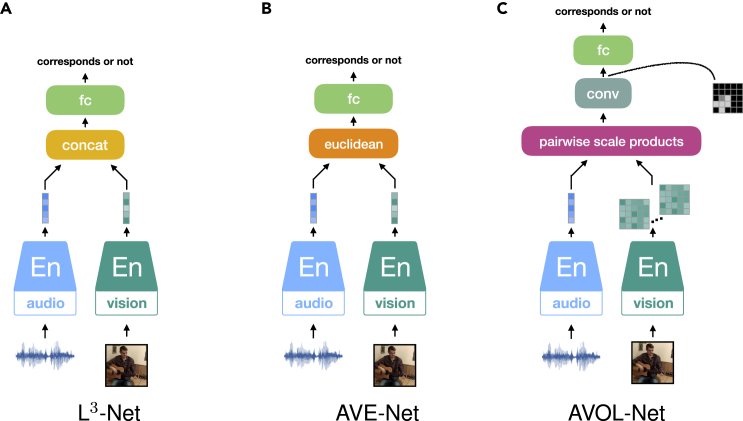
Diagrams for L^3^, AVE-Net, and its modified version—AVOL-Net—for visual object localization

By splitting a video into visual and audio streams, both L^3^-Net^129^ (cf. Figure 9A) and AVE-Net^130^ (cf. Figure 9B) exploit two convolutional networks, named vision sub-network and audio sub-network, to separately encode the two streams into a common space for cross-modal retrieval. Specifically, based on the alignment between both streams, one second of an audio segment and the corresponding center video frame are fed into these two networks. The model is required to decide whether the two inputs are in correspondence or not. For a video clip, the audio segment and the video frame at the same time step are considered as a positive pair for model input, while a negative input pair is the audio segment paired with a video frame from another different video clip. In L^3^-Net,^129^ the audio and visual representations are concatenated before being sent into fully connected layers to predict the correspondence score. In contrast, AVE-Net^130^ measures the correspondence degree by computing the Euclidean distance between audio and visual representations that are designed to be of the same dimensionality. Moreover, both L^3^-Net and AVE-Net are especially designed for recognizing where the sound is generated in the video frame, for example, the location of specific instruments in a band. The vision sub-network of L^3^-Net has the intrinsic ability to recognize semantic entities that make sound, while AVE-Net needs additional modifications on its model architecture, incorporating a comparison mechanism to the audio representation with each spatial grid of the 3D visual representations (cf. Figure 9C). The method encourages at least one region to respond highly for a corresponding audio and video frame and, hence, enables the localization of the object that sounds. As these two visual-audio correspondence (AVC) works formulate the task as a binary classification problem, the models can be optimized by minimizing a logistic loss. For the C^3^ learning method presented in Jansen et al.,^131^ the task to predict the correspondence between audio and image frames is relaxed to a less-restrictive notion of coincidence. Moreover, three auxiliary tasks (ATs), each of them introducing an auxiliary network and an additional loss, are involved in a curriculum way in order to train the audio model while improving its representation capability. After the convergence of AV coincidence loss, a prediction task aimed at only deciding the coincidence of audio pairs (audio-audio correspondence) is performed and jointly trained with the AV coincidence loss. For this, a critical timescale needs to be considered to qualify as coinciding. Afterward, an improved clustering method, entropy-based clustering, is used to categorize the audio representations from the audio embedding network. The model can be further advanced via explicit supervision, but only one label for each cluster is requested given the sufficiently pure clustering results.

In Owens and Efros,^132^ a model to predict whether the visual and audio streams are synchronized is trained, with contrastive objectives, using a sequence of video frames instead of a single frame. Nagrani et al.^133^ suggest applying curriculum learning, i.e., starting the training with relatively easier negative and positive pairs for good model initialization and gradually increasing the difficulty of input pairs for easier model convergence. The approach has shown promising results in learning cross-modal embeddings for the recognition of a person’s identity. However, an incoming problem is that the model tends to rapidly learn to differentiate easy negative pairs from positive pairs, while harder input pairs have very limited effect on learning discriminative representations. An opposite curriculum schedule has been shown to be effective for training a model that learns the cross-modal embeddings for ultrasound.^134^ The visual and audio streams used in this work are medical ultrasound videos and the voice of a sonographer during the video recording. Due to the sparse correlation between the two inputs, hard positive and negative input pairs are first used in order to force the model to learn more strongly correlated representations. In Zhang et al.,^135^ the authors introduced a two-stage curriculum learning solution based on teacher-student training and identified it as self-supervised curriculum learning for audio-visual representation learning. Before joint training of vision and audio sub-networks, one of the sub-networks is updated using the representations from the other sub-network as a teacher, which is frozen for updating. The two sub-networks exchange the role of training with the other sub-network. In addition, to enlarge the number of negative samples for training, a memory bank is applied, resulting in considerable improvements in a visual task of action recognition and an acoustic task of audio sound recognition.

**Figure 10:**
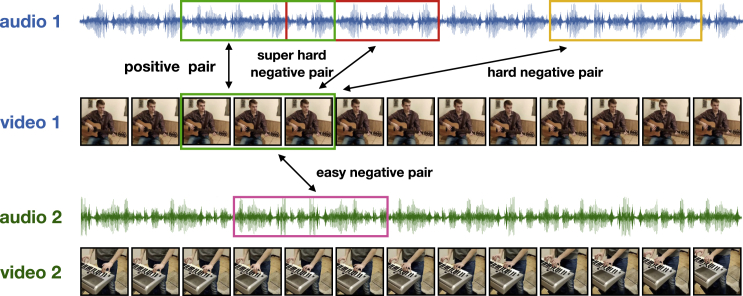
Diagrams for demonstrating a negative sampling strategy The strategy was introduced in Korbar et al.^136^

Differently, in Korbar et al.,^136^ the authors make use of margin loss in order to contrast positive and negative pairs that are of equal proportion. The negative examples of different hardness difficulty are considered, including easy negatives, hard negatives, and super-hard negatives (shown in Figure 10). The easy negatives are video frames and audio segments from different videos, while hard negatives are those pairs taken from the same video but that are at least half a second distant from one other. The super-hard negatives are the pairs that overlap for a certain temporal extent. The authors also confirmed the need to start to train the model with easy negatives and then gradually add harder negatives, which has shown to be effective for learning high-quality representations. Similarly, treating negative pairs of different specialties, i.e., different difficulty levels, is investigated in Ding et al.^137^ for audio-visual speaker diarization. In this work, the margin value used in a triplet loss is controlled by the shifted range between audio and visual streams, thus representing a different difficulty degree of negatives. Nagrani et al.^138^ optimized a model to learn audio-visual representations by formulating negative samples from the same video and different videos in content loss and identity loss. Additionally, in order to encourage explicit separation of representations, they used a disentanglement loss, which is implemented as confusion loss in Alvi et al.^139^

Harwath et al.^140^^,^^141^^,^^142^ proposed another interesting pretext task by associating spoken audio captions with their corresponding image for learning audio-visual representations. Two networks are used to process the audio and image as inputs. In Harwath et al.,^140^ the dot product of a pair of visual and audio representations is calculated as their similarity score. Similar to AVOL-Net, similarity between audio representation and the visual embedding of each pixel can be computed to construct spatial activation maps, leading to a solution for object localization.^141^ In a different way, in Harwath et al.,^142^ the generated audio and visual representations are pushed into a common latent space using triplet loss as well as by contrasting the positive pair to the negative pairs that contain either an unmatched caption or an unmatched image. Hsu et al.^143^ solve the same task by building a system based on ResDAVEnet-VQ^142^ and an image-to-unit model.^144^ Although each of these models is used to process an input stream, the two representations are pushed into the same latent space. Instead of using contrastive training objectives to reproduce the audio input, the learned discrete linguistic units, learned through ResDAVEnet-VQ from the input utterances, are fed to Tacotron2,^145^ i.e., a text-to-speech (TTS) model for speech synthesis. The visual sub-network, i.e., ResDAVEnet-VQ, is then expected to learn representations that are close to the discrete linguistic units, thus enabling representation learning to retrieve information from both modalities.

**Figure 11:**
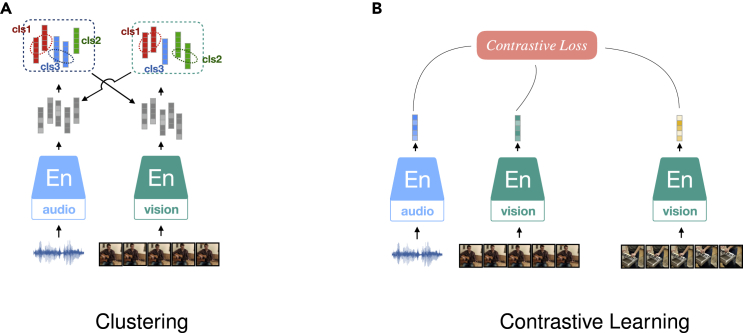
Methods for audio-visual mutual supervision

Based on their mutual correspondence, the audio and visual streams of a video clip can be seen (to each other) as the supervisory signal for representation learning. An earlier study has shown the success of using synchronously recorded ambient sounds as supervision for visual learning.^146^ Later on, Alwassel et al.^147^ and Morgado et al.^148^ empirically verified that exploiting the representation of one modality to create pseudo-labels for training the encoder network of the other modality outperforms not only SSL on a single modality but also SSL based on pseudo-labels of both modalities. In Alwassel et al.,^147^ the pseudo-labels are generated using a deep clustering method (cf. Figure 11A). Differently, Morgado et al.^148^ aggregates “memory features” by computing the slow EMA and subsequently applies contrastive learning (cf. Figure 11B), similar to Grill et al.^49^ In Morgado et al.,^148^ cross-modal agreement (CMA) is additionally introduced to enhance the interactions between instances, specifically to calibrate within-modal similarities between positive pairs. Both methods, i.e., clustering- and contrastive-learning-based modeling, learn effective audio-visual representations, evaluated on a downstream task of action recognition based on video. For the same downstream task, Morgado et al.^149^ further address the robustness in learning audio-visual representation learning, considering two noisy input cases: faulty positives and faulty negatives. The effect of faulty positives, representing the audio and video signals that are of scarce information for each other, are alleviated by assigning less weights in the overall contrastive loss. Faulty negatives indicate the sampled negatives that are semantically similar to the base instance; this is tackled by estimating softening scores between the base instance and negatives instead of assuming that every negative instance is “equally negative.”

Incorporating spatial cues occurring in audio and video streams, learning audio-visual representations can be extended to be from 360° video and spatial audio.^150^ This is done by performing audio-visual spatial alignment (AVSA) in contrastive learning, where negative samples are audio and video clips generated from different viewpoints within a 360° video, and spatially misaligned audio and video pairs. Alternatively, in Masuyama et al.,^151^ multi-channel audio is associated with the candidate direction of arrivals (DoAs) estimated using a visual network from 360° video.

#### Audio-visual source separation

**Figure 12:**
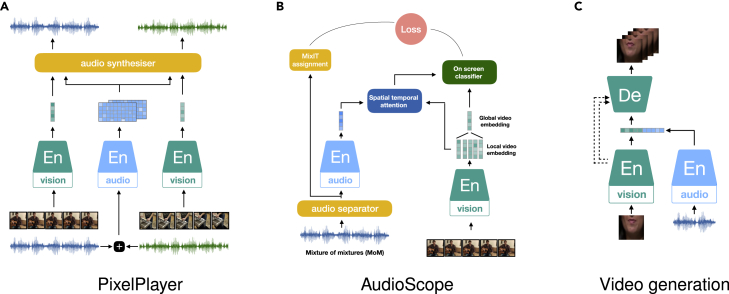
Diagrams for PixelPlayer and AudioScope and the framework for video generation

The PixelPlayer^152^ effort proposed a mix-and-separate framework that solves visual object segmentation and audio source separation together. The framework consists of three networks: a video analysis network, an audio analysis network, and an audio synthesizer network, as shown in Figure 12A. The video analysis network extracts visual features from a sequence of video frames, while the audio analysis network processes the mixture sound from two different video clips. The audio synthesizer network aims to separate the audio sources based on the learned audio representations of the mixture, conditioned on the corresponding video frames. In this way, the model can learn a better semantic visual representation that is highly associated with its own audio but is less relevant to the audio of the other video clips. Although the learned audio representations enable the model to retrieve information from the mixture sound, it cannot separate audio from each video. In a later work, the same authors also indicate that having synchronized audio and visual data is a requirement to disentangle the learned audio and visual representations before feeding them into the audio synthesizer network.^154^ By doing this, the learned audio and visual representations can be used independently.

**Figure 13:**
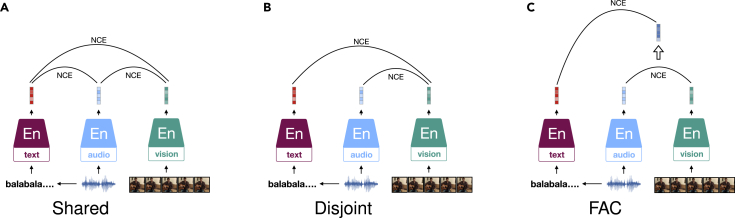
Diagrams for three modes in a multi-model versatile network The network was based on Rouditchenko et al.^153^

AudioScope^155^ expands the conditioning audio separation approach and exploits an additional audio embedding network to process the separated audios. An audio representation then aggregates the global information from each resulting audio embedding using temporal pooling. Subsequently, attention is used to retrieve the mutual information between the local spatial-temporal video embedding (learned with the video embedding network) and the global audio representations. This allows us to generate an audio-visual representation that combines the audio and visual information. Finally, the audio-visual representations, i.e., global video embedding and global audio embedding, are concatenated together. By this, based on the separated audios, it is expected to create the MixIT assignment.^125^

LWTNet^156^ designs a model that can ingest a video and transform it into a set of discrete audio-visual objects using SSL. Similarly, an audio network and a video network encode the audio and video frames; then, a fine-grained audio-visual attention map is computed by solving a synchronization task, i.e., measuring the similarity between the audio and the visual features at every spatial location. The model can detect and track multiple audio-visual objects and extract an embedding for each of them. Given negative audio samples from shifted audio segments of the same video clip, contrastive loss is applied to maximize the similarity between a video frame and its true audio track. This, which is made in the form of an attention map, also minimizes the similarity of the misaligned versions of the audio.

#### Video generation

Given a starting video frame of a speaker, previous work has shown that a model can be trained to generate the subsequent video sequences based on the corresponding speech utterance.^157^^,^^158^^,^^159^ In these works, a U-Net is used to code the starting video frame into a latent representation, while an audio encoder is used to learn a representation of the speech utterance. In Shukla et al.,^157^ the visual and audio representations are concatenated and then fed into the decoder of the U-Net to generate the full sequence of video frames. Using neural decoders, the raw waveform, the log mel spectrogram, and the MFCC spectrogram of the audio input are expected to be constructed from the learned audio representation. L1 reconstruction errors between the output of the decoders (both audio and video) and the ground truth are minimized. Differently, in Shukla et al.,^158^ the use of audio decoders is avoided, and the length of the input audio is reduced to 0.2 s. In this work, a vector randomly created using Gaussian noise is appended to the audio-visual representation, thus injecting randomness in the procedure of face synthesis. In Shukla et al.,^157^^,^^158^ the authors show the efficacy of these two methods for spoken word classification and lip-reading tasks. A later approach by the same authors is also developed for emotion recognition.^159^

BraVe^160^ exploits the framework of BYOL, where the two sub-networks process a short time frame in a video (about 1–3 s), known as a narrow view, and the other one handles a larger extent of the video, known as a broad view. By minimizing the loss of BYOL with respect to each sub-network, the model is essentially trained to predict a broad view from the narrow view and to regress the narrow view from the broad view. Instead of using only video as the supervisory signal for training the model, using audio as well as a combination of visual and audio broad views is also possible for the prediction task.

### Audio and text

Baevski et al. present wav2vec unsupervised (wav2vec-U),^161^ which learns a mapping from audio representations to phonemes directly without supervision. The method is a GAN where the generator uses wav2vec 2.0 to extract speech representations, and based on this, it generates a phoneme sequence using a clustering method. The generated phoneme tries to cheat a discriminator that is conditioned on a real phoneme sequence from unlabeled text. Similarly, Chung et al.^162^ proposed learning the individual speech and text embedding spaces by aligning the two spaces via adversarial training and subsequently applying a refinement procedure. Under the assumption that embedding spaces from two modalities share a similar structure, in this work, the audio and text embeddings are first learned using Speech2Vec and Word2Vec, respectively; then, adversarial training is used in order to learn a linear mapping between the speech embedding space and the text embedding space. The approach has shown promising results for the task of ASR and speech-to-text translation systems for low- or zero-resource languages such as German, which has little audio-text pairs for training.

The coalignment of audio and text can also be done within an auto-encoder architecture. COALA, presented in Favory et al.,^163^ applies two AEs to process an audio spectrogram and the audio tag. Both AEs are optimized to reconstruct its input, resulting in semantic features of the audio and the text. The paired semantic features are pulled closer, and the unpaired semantic features are pushed further, using a contrastive loss. The whole system is jointly optimized by minimizing the two reconstruction errors and the contrastive loss as a multi-task learning problem. Affine transformations are applied to the two learned representations, reducing the difficulty in maximizing their agreement. An auto-encoder is also used in Haque et al.^164^ for encoding audio spectrograms. As in COALA,^163^ the latent representations are also expected to be able to reconstruct the spectrogram and predict the linguistic features simultaneously. In these two works, one modality, either text or audio, is used to learn an embedding that is used to predict its paired input (in the other modality). Hence, it can be assumed that the learned embedding contains the information from both streams. The reconstruction can be seen as a regularization term that enables the embedding to reconstruct the input stream, thus ensuring that the learned latent contains the salient features of the input stream. Similarly, CSTNet^165^ is trained for speech translation, but speech utterances are in English, while the text translations are in any other language from French, German, Spanish, Portuguese, or Italian. The experimental results obtained from CSTNet indicate that the speech representation learned using this framework can achieve comparable results for two downstream tasks, i.e., a minimal-pair ABX task and phone recognition.

### Audio, text, and video

To conclude this section, we will introduce some SSL works that learn audio representations through the use of three modalities: video, audio, and text. In these works, texts are commonly achieved by using off-the-shelf ASR systems from audio. For instance, in Sun et al.,^166^ the authors presented the use of keyword localization as the pretext task. The authors also compare the performance obtained by separately using text or images as the supervisory signal. They conclude that the visually supervised model performs worse than a text supervised model based on BoW. Indeed, although the visually trained model can sometimes locate semantically related words, this phenomenon is not consistently observed.

A multi-modal versatile network is presented in Alayrac et al.,^153^ a study that aimed to find the best combination of the modalities (refer to Figure 13). Learning a shared space of the three modalities, as well as two separate disjoint spaces for video-audio and video-text (considering that text originates from the audio), are investigated. Fine and coarse (FAC) spaces are additionally proposed due to the fact that the visual and audio domains differ (in terms of granularity) with respect to the language domain. In FAC, vision and audio are compared in a fine-grained space, while text, audio, and vision are compared in a lower-dimensional coarse-grained space. For this, the visual representation is first mapped into common latent spaces of audio and video and sequentially projected into the common latent spaces of text and audio-visual common spaces. The authors consider no direct link between audio and text. Similar to the FAC approach, VATT^167^ also presents a two-stage multi-modal projection. In VATT, audio and video are compared first using NCE loss. Subsequently, through the use of multiple-instance learning (MIL)-NEC loss^153^ for optimization purposes, the text is included in order to learn common latent spaces for the three modalities. Moreover, the authors suggest using transformers for encoding all three modalities, which leads to a more uniform but efficient architecture.

## Downstream audio tasks and benchmarks

After solving pretext tasks, an audio SSL model is expected to produce high-quality audio representations that are of sufficient generalization and discrimination, thus guaranteeing a good performance on downstream tasks. Several different downstream audio tasks have been considered for empirically measuring the audio representation quality. For example, ASR is used for evaluating all wav2vec-based methods.^72^^,^^77^^,^^78^ Other tasks include speaker identification (SI),^73^^,^^86^^,^^168^ speech emotion recognition (SER),^169^^,^^170^^,^^171^^,^^172^ speech machine translation (SMT),^173^ pitch detection (PD),^174^ ASC,^92^ and music classification,^175^ among others.

In Table 3, we summarize the information of some publicly available benchmarks that enable fair comparisons between different audio SSL algorithms. Most of these benchmarks concentrate on speech-related downstream tasks. One prominent pioneer benchmark is the zero-resource speech challenge (ZeroSpeech) (https://zerospeech.com),^176^ which has addressed all aspects in building an end-to-end spoken dialog (SD) system. The first challenge started in 2015 (ZR15) with two task tracks of unsupervised discovery of linguistic units, on different levels of linguistic structure, from raw speech in an unknown language. The first task track targets the investigation of unsupervised sub-word modeling methods that produce a speech representation robust to within- and between-speaker variation. The second presents the task of spoken term discovery and audio word segmentation, which split the “words” in an input raw speech using unsupervised-learning approaches. The tasks aimed at acquiring proper acoustic modeling and lexicon generation. The requirement of these two tasks were raised in 2017 in order to better handle language variants rather than just speaker variants. For the first task, ABX scores (within and across speakers) are computed directly on the learned representation in order to evaluate its quality. Specifically, this is achieved by computing the average of the frame-wise cosine distance of the representations of the tokens along the dynamic time wrapping (DTW)-realigned path. For the second task, a total of 17 evaluation metrics were considered for measuring each step of spoken term discovery.^177^ The tasks of ZR19 and ZR20 were to address an additional problem of speech synthesis without any text or phonetic labels. In addition to discovering sub-word units given as raw audio, the participants were also supposed to align the units to the voice recording (as well as possible) for the purpose of re-synthesizing utterances of target speakers. Low-bit rate but high quality in the representation of linguistic units, measured based on ABX scores, were expected for the discovery step. The synthesis accuracy was supposed to be calculated using three measures, which needed human assistance, i.e., character error rate (CER) between the human transcription of synthesized speech and the gold transcription, mean opinion score (MOS), and similarity to the target voice. The latest challenge, launched in 2021, provided several tasks for spoken language modeling based on speech only as well as visually grounded language modeling. Speech-based language modeling consists of learning language models directly from raw audio in an unknown language. Visually grounded language modeling aims at learning language models by incorporating the visual information. The performance can be measured with respect to four linguistic levels, i.e., phonetics, lexicon, syntax, and semantics.

**Table 3:** An overview of the benchmarks for evaluating audio self-supervised learning methods

Benchmark	Audio domains				Tasks	SOTA method
	Speech	Env.	Music	Semantic		
ZeroSpeech^176^	✓	–	–	✓	sub-word modeling, STD, SSyn, spoken language modeling	–
SUPERB^118^	✓	–	–	✓✗	ASR, PR, SCR, query by example STD, SI SV, SD, intent classification, slot filling, SER	WavLM^113^
SUPERB-SG^117^	✓	–	–	✓	SUPERB tasks + ST, out-of-domain ASR, VC, SE, SS	–
LeBenchmark^178^	✓	–	–	✓	ASR, SLU, ST, SER	–
Libri-Light^179^	✓	–	–	✓	ASR	–
NOSS^76^	✓	–	–	✗	SER, SI, LI, medical diagnosis	–
HEAR^180^	✓	✓	✓	✓✗	SCR, PD, SED, IC, SER, ASC, MTrans, LI, MGC, SCI, AT, and others	–
HARES^181^	✓	✓	✓	✓✗	AT, AniSC, ASC, SCR, LI, SI, IC, PD, MT	Slowfast NFNet-F0^181^

Name, involved audio domains (i.e., speech, environment, music), semantic (i.e., semantic or non-semantic representations), involved tasks, and the SOTA method, when applicable, are given. ASR, automatic speech recognition; PR, phoneme recognition; SI, speaker identification; SV, speaker verification; SER, speech emotion recognition; SE, speech enhancement; SS, speech separation; ST, speech translation; SD, speaker diarization; VC, voice conversation; SSyn, speech synthesis; STD, spoken term detection; SCR, speech command recognition; LI, language identification, PD, pitch detection; AT, audio tagging; ASC, acoustic scene classification; SED, sound event detection; AniSC, animal sound classification; MT, music tagging; IC, instrument classification; MGC, music genre classification; SCI, speaker count identification; MTrans, music transcription.

The speech-processing universal performance benchmark (SUPERB) (https://superbbenchmark.or^a^https://generallyintelligent.ai/blog/2020-08-24-understanding-self-supervised-contrastive-learning/g/)^118^ presents a standard and comprehensive testbed for evaluating audio representations that consists of 10 tasks focusing on linguistic, shallow semantic, speaker, and prosodic characteristics. Its later version, i.e., SUPERB-SG,^117^ extends the evaluation methods by five additional audio tasks aimed at examining the semantic and generative capabilities of audio SSL models. These tasks are speech focused, covering the purposes of recognition, conversion, separation, translation, and enhancement, all of which require the learned representations to be versatile in capturing linguistic, semantic, and speaker characteristics. SUPERB and SUPERB-SG exploit the widely applied evaluation metrics for each considered task. For example, accuracy is used as the performance measure for all the classification tasks involved. Word error rate (WER) and phone error rate (PER) are used to evaluate the performance of speech recognition and phoneme recognition. The detection task of query by example spoken term detection can be effectively measured using maximum term weighted value (MTWV), an evaluation metric balancing misses and false alarms. As performance measures of speaker diarization and verification, diarization error rate (DER) and equal error rate (EER) are used. For the task of slot filling, both slot types and slot values are considered to be important for building a spoken language understanding (SLU) system, while F1 score and CER are separately adapted to evaluate each aspect. For the 10 SUPERB tasks, WavLM dominates the state-of-the-art results in the leaderboard. SUPERB-SG also considers standard evaluation metrics for measuring the performance of downstream tasks. The task of speech translation (ST) aims to translate speech signals from a source language into another and can be evaluated using sacreBLEU and the case-sensitive de-tokenized BLEU.

LeBenchmark (http://lebenchmark.com/)^178^ is another reproducible and multi-faceted benchmark providing four downstream tasks for evaluating speech SSL models for the French language. For reproducibility, the LeBenchmark organizers provided pre-trained SSL models learned on different sub-sets of a large and heterogeneous collection of read, prepared, and spontaneous speech utterances in French. Different from the discrete emotion classification task presented in SUPERB, LeBenchmark involves a SER task for continuous emotion status represented by arousal and valence. For this, concordance correlation coefficient (CCC) of emotion predictions is computed to measure the performance.

Libri-Light^179^ is a benchmark specifically designed for the task of ASR with limited or no supervision. Libri-Light is based on spoken English audio collected from open-source audio books of the LibriVox project.

Focusing on the evaluation of non-semantic speech representation, NOSS^76^ presents a set of paralinguistic tasks, including SER, SI, LI, and medical diagnosis from speech. In particular, SER and speech command recognition are additionally suggested for measuring the speech representations generated from the personalized models, which are trained and evaluated for a specific speaker.

Two benchmarks that can be used to develop and examine universal audio representations across all three (roughly categorized) audio domains, i.e., speech, environmental sounds, and music, are holistic evaluation of audio representations (HEAR) (https://neuralaudio.ai/hear2021-holistic-evaluation-of-audio-representations.html)^180^ and holistic audio representation evaluation suite (HARES).^181^ HEAR requests participants to create an audio representation that is as holistic as the human ear, and the benchmark contains nineteen diverse downstream tasks. HERAS unifies 12 existing datasets spanning multiple audio domains. Accuracy is used to evaluate the classification tasks, while the performance of the tagging tasks involved, like audio tagging and music tagging, which aim to predict multiple classes at output, are measured using mean average precision (mAP).

For audio-visual SSL, experiments for pretext tasks are typically based on several public available datasets, including AudioSet,^182^ SoundNet,^183^ Kinetics-400,^184^ VoxCeleb1/2^185^^,^^186^, or lip reading in the wild (LRW),^187^ to cite a few. The recently proposed ACAV100M^188^ is an automatically curated dataset of 100 million 10 s clips carefully chosen from a total of 140 million full-length videos, thus solving the optimization problem to maximize the mutual information between audio and video. This dataset and its sub-sets (also provided with different scales) (https://acav100m.github.io/) supply large-scale data of high audio-visual correspondence, and therefore learning high-quality audio-visual representations is foreseeable.

## Discussion

In this section, we first clarify the differences and similarities between SSL methods and other confusing machine-learning mechanisms. Next, we discuss the common problems and difficulties met during the development of SSL models. We further point out some additional concerns regarding audio SSL, considering the difference in data processing and augmentations, negative sample generation, and network construction, compared with SSL approaches for other modalities.

### Difference from other confusing learning mechanisms

Generally speaking, representation learning aims to capture the posterior distribution of the underlying explanatory factors from the observed input data. A good representation should be of sufficient generalization and distinctiveness so that it carries complete salient information of the data that is useful as input for supervised tasks, such as classification. Representation SSL is a representation-learning approach that trains a model in order to produce representations. This is achieved by solving specially defined pretext tasks based on, usually very large-scale, data without human annotations. This is different from the classic learning mechanisms of transfer learning and domain adaptation, which learn to generate representations in supervised frameworks, i.e., using labeled data. SSL is commonly regarded as an unsupervised-learning method, as like the ones using data without human annotations. However, it is also different from classic unsupervised learning, such as clustering, because these kinds of unsupervised learning concentrate on grouping inputs that have similar data patterns, whereas SSL learns representations with supervision of some automatically created training targets, such as pseudo-labels. Likewise, it is considered unsupervised in the sense that no labels from the target task are involved.

Contrastive SSL is highly related to distance metric learning (or simply, metric learning).^189^ Given an anchor paired with positive samples and negative samples, weakly supervised metric learning constructs a distance metric that puts positives close together and negatives far away in a latent space. Hence, contrastive SSL can be seen as a metric learning where the positive pairs are created from the same data source through procedures such as DA. Contrastive SSL is also similar to instance discrimination.^190^ Instead of processing positive and negative pairs, instance discrimination takes each data sample as from a separate class and learns feature representation that discriminates among individual instances. According to our analysis of Equation 5, when the temperature parameter is set too small, the InfoNCE loss tends to take the two inputs of a positive pair as the different instances and optimizes the SSL model using the method of instance discrimination.

Besides, generative adversarial networks (GANs) are also seen as a kind of SSL framework in some works.^8^^,^^11^^,^^17^ For instance, the generator creates data from a random vector by taking the real data as training targets. Then, the discriminator network aims to measure the similarity between generated and real data. It is worth noticing that the similarity measure changes as the discriminator is updated. Such a kind of generative contrastive model has been successfully investigated for NLP tasks, such as in ELETRA,^191^ but has rarely been explored for audio SSL. Hence, we did not introduce it as an audio SSL form in the literature review, though it should be naturally considered for future works.

### Difficulties and problems for SSL optimization

The representation quality using SSL is determined by the efficacy of pretext tasks, of which the key component is the design of training targets or objectives. The training objectives for both predictive and contrastive SSL concentrate on the correlations between representations of observed data. Both methods concentrate on maximizing the similarity between the representations of the two views of one unique data sample. Additionally, contrastive methods contrast the similarity against the distance to other data samples.

Representational collapse often appears when training a predictive SSL model, such as using a Siamese network architecture. To tackle this issue, the pair of networks is usually designed to be of asymmetric architecture and is updated asynchronously. DA techniques, used to generate different views of input data, are used to additionally force the Siamese network to process asymmetric input. Contrastive SSL alleviates the problem of mode collapse by driving the representations of samples, including positives and negatives, to maximal-uniformly distributed appearance on a unit sphere. Minimizing a contrastive loss, such as InfoNCE, is found to be approximately equivalent to maximize the mutual information between representations. With the rise of the number of negative samples, a lower bound on mutual information is raised up.^17^^,^^42^ Therefore, better representations that carry more correlation information between representations can be obtained by enlarging the amount of negative samples, for instance, as shown in Chen et al.^15^ and He et al.^29^ Similar considerations have led to the success of contrastive audio SSL.^42^^,^^72^^,^^77^ For this to happen, however, the requirement of memory dramatically boosts. Therefore, negative sampling of better efficiency needs to be further explored. On the other hand, according to the theoretical analysis in Saunshi et al.,^18^ a too-large number of negative samples may not be profitable for training contrastive SSL models. So far, no research has been done to suggest a golden standard rule for setting a proper number of negatives. Moreover, the setting should potentially be considered differently for different tasks and applications. Another issue that can hamper contrastive SSL is early degeneration,^11^ which means that the SSL model over fits to the discriminative pretext task in very early training steps, and therefore, the representations do not present a sufficient generalization ability. Solutions that can relax this early degeneration issue should also be addressed in future work.

### Additional adjustments on SSL for audio

As introduced above, SSL approaches that have been well explored for CV and NLP tasks are being transferred to the audio domain. For this, some works process 1D audio data into a 2D format in order to match the formulations of these SSL frameworks. For example, time-frequency representations of audio and advanced transformations based on it, such as a spectrogram, mel spectrogram, and MFCC, can be used as images in some SSL models designed for CV tasks.^74^^,^^83^^,^^96^ For this case, DA techniques widely used in the CV domain have also been considered, which are essential for achieving high-quality representations. Taking the features as sequential frames, we can process them in similar ways as we would do for NLP tasks.^89^^,^^90^

An alternative way is to directly process the 1D waveform using deep-learning encoders, such as 1D convolution, which converts the 1D signal into higher-dimensional features for further processing. This solution has been successfully used in Oord et al.,^42^ Baevski et al.,^72^ and Pascual et al.^85^ The focus of this review was not to assess network architectures but rather to concentrate on the framework and formulations of SSL approaches. In most visual SSL works, the research concentrates more on the formulations rather than the network architecture, for which a ResNet is typically used. However, the importance of neural network architectures is not that clear for audio SSL. Researchers tend to use network architectures that were designed to respect the speech or audio structure, which can achieve more promising results in the context of audio SSL. A recent work has explored the influence of different neural network architectures in learning universal audio representation.^181^ Still, more research evaluating the effect of network components, such as assessing the effect of the attention mechanism used in transformers,^97^ should be carried out.

### Fitness and mismatch between pretext and downstream tasks

In general, we expect that by training a model with SSL, it is possible to learn general representations that are effective for downstream tasks. Although this is slightly different from classic transfer learning, which performs pretext tasks in a supervised framework, the gap between the source data in pretext tasks and target data for downstream tasks is expected to be matched.

Comparing speech and other audio signals, such as acoustic scene recordings, the speech signal is more variable from a temporal and frequency perspective, while the acoustic scene recordings are usually more stationary along the temporal axis. Hence, different kinds of pretext tasks need to be considered in order to retrieve the acoustic features that are discriminative in terms of global information or transient information. For downstream tasks concerning non-semantic features from speech, methods’ proficiency in extracting both global and transient information has been explored.^76^

In the standard framework of SSL, labeled data are used in downstream tasks for fine-tuning. It has been shown that a small quantity of labeled data can already guide a pre-trained model to achieve very satisfying performance on downstream tasks. This inspired semi-supervised learning using very little human-labeled data from the target data domain to close the gap between source and target data. Specifically, the training objectives of SSL and supervised learning are combined and optimized simultaneously. For many audio applications, SSL approaches have shown promising performance and reached (or even surpassed) state-of-the-art results achieved through supervised learning. Still, when labels are available or partly available, like in CLAR^81^ and UniSpeech,^192^ combining SSL and SL together into a multi-task learning setting enables to learn better speech representations for some audio tasks.

### Conclusion

This survey has provided an overview of the existing approaches and methods for uni-modal and multi-modal SSL approaches using audio. The success of these methods has been analyzed in several classic audio tasks, including speech recognition, SI, SER, and ASC. Audio SSL methods, such as wav2vec 2.0 and HuBERT, have been shown to even surpass the performance of supervised-learning methods on the same task. Moreover, the generalization ability of representations learned using audio SSL can decrease the urgency of searching for hand-crafted, engineered features. The superior performances obtained using SSL-based approaches support the generalization capabilities of this representation-learning method and encourage the use of this technique to shape the future and advance the state of the art in the field of audio processing.
